# Scaling Gaussian Processes for Deterministic Model Predictive Control with Online Updates: A Framework for Model Selection and Re-identification in Process Control

**DOI:** 10.1021/acs.iecr.6c01228

**Published:** 2026-08-14

**Authors:** Michael W. Fouts, David S. Mebane, Fernando V. Lima

**Affiliations:** † Department of Chemical and Biomedical Engineering, 5631 West Virginia University, Morgantown, West Virginia 26506, United States; ‡ Department of Mechanical, Materials and Aerospace Engineering, West Virginia University, Morgantown, West Virginia 26506, United States

## Abstract

Gaussian processes (GPs) are powerful, nonparametric models, widely recognized as universal function approximators due to their ability to provide robust probabilistic predictions alongside quantified uncertainty estimates. This has allowed for the modeling of complex processes in chemical engineering with applications to process optimization, prediction, and control. However, the practical adoption of standard GPs is severely constrained by their computational training complexity $O\left(n^{3}\right)$ . This bottleneck is particularly problematic in modern high-throughput environments, such as those associated with Industry 4.0, big data applications, and advanced manufacturing. Addressing this demand requires the implementation of highly efficient online learning and model updating strategies. This paper specifically looks at deterministic nonlinear Model Predictive Control (MPC) and proposes a framework for efficiently identifying a highly performant Gaussian process approximation model and re-identification metric, alongside stability and feasibility analyses. The effectiveness of these techniques in a real-time system is demonstrated through a cascaded tanks experiment.

## Graphical abstract

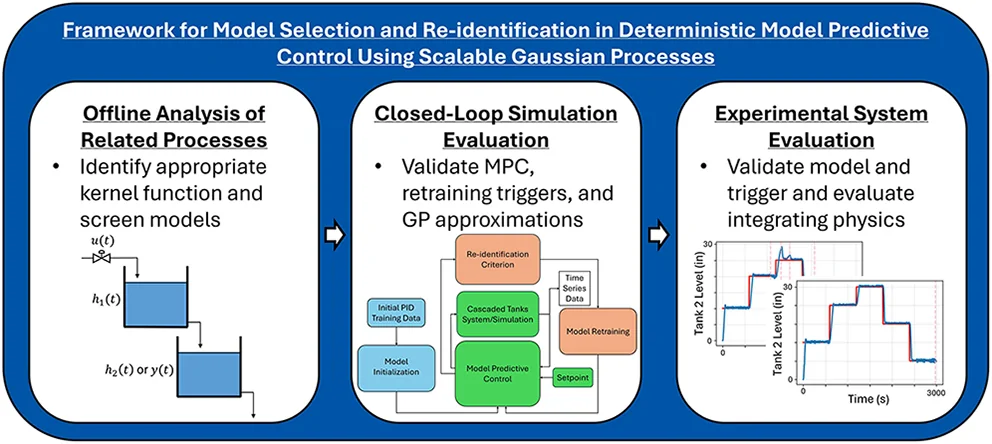

## 1 Introduction

The chemical engineering discipline is undergoing a significant transition influenced by advancements in computing, automation, and the convergence of technologies like the Internet of Things (IoT) and Industry 4.0. This digital transformation results in the generation and collection of massive amounts of data. The continuous influx of big data offers new possibilities for increasing the information gained from chemical engineering data sets, leading to novel solutions for improving operational reliability and efficiency.^1–5^

Traditionally, chemical engineering has relied heavily on first-principles mechanistic models derived from fundamental laws such as mass and energy balances. However, for increasingly complex industrial processes, deriving, simplifying, or executing these rigorous mechanistic models can be knowledge-intensive and computationally demanding. The availability of massive data sets now facilitates the use of alternative data-driven models that rely on process data to infer patterns and knowledge with minimum a priori assumptions about the underlying mechanism. This paradigm shift provides critical insights for process improvements and enables new approaches to data-driven operations.^1,3,6^

Among the data-driven techniques, Gaussian processes (GPs) are highly popular nonparametric Bayesian models frequently used for modeling, surrogate optimization, and function approximation.^7,8^ A key strength of GP models is their ability to yield a probabilistic predictive distribution, providing robust predictions coupled with a crucial quantification of uncertainty (variance). While this work uses the mean prediction for a deterministic GP-MPC implementation, the ability to estimate confidence in predictions is a unique advantage for developing robust process strategies.

Despite their conceptual advantages, the practical adoption of standard GPs is severely constrained when applied to big data environments. The fundamental reason for this lies in the high computational demands associated with their implementation, particularly the necessary inversion of the dense covariance matrix during training, resulting in a time complexity of $O\left(n^{3}\right)$ . This computational bottleneck prevents the standard implementation of a full GP from being scalable when the training size *n* exceeds $O\left(10^{4}\right)$ data points.^7,9^ This has led some practitioners to choose machine learning options that scale better with data, such as neural networks, but at the same time loose the ability to produce inherent uncertainty predictions.^10^

A further challenge specific to industrial chemical processes is the nature of the acquired data. Although high-volume data is generated, it often corresponds to normal, routine operating points or periods of stability (steady-state), leading to data sets characterized as low variance, high volume. This low variability results in much of the collected data being highly redundant or “information-poor”. While techniques that combine chemical engineering and machine learning knowledge aim to increase the information gained from the available data, the sheer size and redundancy of the data sets worsen the $O\left(n^{3}\right)$ scaling problem.^4^

The complexity of modern chemical and energy processes necessitates the use of data driven models. However, process systems are inherently subject to changes (continuous changes such as fouling, event changes such as equipment replacement, and process changes like set point changes). These changes frequently lead to plant-model mismatches. To maintain model performance and address these time-varying systems, the predictive models must be updated quickly and efficiently in an online fashion.^4,11^ To overcome the inherent $O\left(n^{3}\right)$ computational scaling issue, which prohibits updating a full GP online, researchers have developed various scalable GP approximation methods which fall into one of three methodologies: Sparse GPs (global approximations or inducing points), localized GPs, and decomposed GPs.^12–15^

To handle the dynamism of a changing process, systems rely on evolving re-identification strategies, which iteratively adapt both the structure and parameters of the predictive model using new data to address time-varying systems. Historical metrics for identifying model retraining vary in logic including looking at predictive uncertainties to identify sparse regions of training data and comparing model/prediction mismatches to identify poor performing regions.^11,16–18^ However, no studies have compared these retraining triggers to determine how differences in their responses affect model performance.

This paper builds upon past work^18^ to address questions raised during practical implementation of GPs in scalable online environments, specifically, the selection of the GP model and choice of re-identification criteria. The primary contribution of this work is a systematic framework designed to efficiently identify highly performant model architectures and re-identification metrics for controls implementations. This encompasses validation with offline data, online synthetic environments, and real-world experiments to minimize development effort while maximizing rigorous model assessment. Through this evaluation, the framework provides a novel comparative analysis of the practical trade-offs between Gaussian process approximation techniques and re-identification metrics within an MPC context, addressing a gap in the current literature. A detailed description of the proposed methodology is presented in Section 3, followed by the results of its application to the cascaded tanks benchmark problem in Section 4.

## 2 Background

### 2.1 Gaussian Process Regression

GPs are a powerful class of probabilistic models widely used for regression, classification, and other machine learning tasks. They are a generalization of multivariate Gaussian distributions to infinite dimensions, where each point in the input space is associated with a normally distributed random variable. GPs are particularly useful for modeling nonlinear functions and capturing the uncertainty associated with them.

A Gaussian process is a collection of random variables, any finite number of which have a joint Gaussian distribution. Formally, a GP is defined by a mean function *m*(**x**) and a covariance function *k*(**x**, **x**′), as shown in eq 1, such that

1 $$m\left(x\right)=E\left[f\left(x\right)\right],\;k\left(x,x^{\prime }\right)=E\left[\left(f\left(x\right)-m\left(x\right)\right)\left(f\left(x^{\prime }\right)-m\left(x^{\prime }\right)\right)\right]$$

where *f*(**x**) is a random function drawn from the GP. The mean function represents the expected value of the function at any point **x**, while the covariance function encodes the correlation between function values at different input points.^19^ The largest weakness of GPs is the cubic time complexity of training $\left(O\left(n^{3}\right)\right)$ due to the inversion of the covariance matrix, which can limit the application to large scale data sets.

A key choice in modeling accuracy of the GP is the selection of the kernel, also known as the covariance function. This determines the properties of the resulting sampled functions and gives a GP its characteristic behavior. Common covariance functions are shown in eqs 2–4.

- Exponential kernel^20^
2 $$k\left(x,x^{\prime }\right)=\sigma^{2}\exp\left(-\frac{∥x-x^{\prime }∥}{l}\right)$$
- Squared Exponential (SE) or Radial Basis Function (RBF) kernel^20,21^
3 $$k\left(x,x^{\prime }\right)=\sigma^{2}\;\exp\left(-\frac{∥x-x^{\prime }{∥}^{2}}{2l^{2}}\right)$$
- Matern kernel^20,22^
4 $$k\left(x,x^{\prime }\right)=\sigma^{2}\frac{1}{\Gamma \left(\nu \right)2^{\nu -1}}{\left(\sqrt{2\nu }\frac{∥x-x^{\prime }∥}{l}\right)}^{\nu }K_{\nu }\left(\sqrt{2\nu }\frac{∥x-x^{\prime }∥}{l}\right)$$

In these kernels, σ^2^ is the function variance, l is the length scale that controls the correlation decay or how quickly the function varies over a larger, global distance, ν is a smoothness parameter that controls the differentiability of the function at a local level, and $K_{\nu }$ is a modified Bessel function. The SE kernel is a popular choice because of its simplicity and the easy interpretability of its length scale parameter, which controls the distance over which inputs are considered correlated. Here, a small value for l suggests that points that are close together can have very different function values, resulting in functions that fluctuate quickly with a high degree of local variation, but are still infinitely differentiable. However, the Matern kernel is generally a more preferred choice because of its flexibility through the tuning of ν. This allows the Matern kernel to model nonsmooth behavior by determining the level of differentiability of the function. If ν = 0.5, the function is not differentiable and can produce very jagged, rough functions and is equivalent to the exponential kernel. However, as ν → ∞, it becomes infinitely differentiable (much smoother) and equivalent to the SE kernel.^19,20^

### 2.2 Sparse Gaussian Processes (Inducing Points)

To address the challenge of the high training time complexity, methods involving inducing points (also known as pseudo-inputs) were developed to formulate computationally tractable approximations, resulting in sparse Gaussian processes (SGPs). The core idea is to introduce a small, fixed set of *M* fictitious input locations, **Z** = {**z**_1_, ..., **z**_*M*_}, and their corresponding latent function values, **u** = *f*(**Z**). These *M* points act as a compressed bottleneck or summary of the full *N* data points, where the key assumption is that the full function posterior, *p*(*f* |**X**, **y**), can be adequately approximated by conditioning only on the function values at the inducing points, **u**.^9,23–26^

By introducing the inducing points, the computation shifts from inverting the large *N* × *N* covariance matrix **K**(**X**, **X**) to primarily dealing with the much smaller *M* × *M* covariance matrix, **K**(**Z**, **Z**). The approximation essentially projects the full data set onto the inducing points, leading to a computational complexity for training and inference that scales as $O\left({\mathrm{NM}}^{2}+M^{3}\right)$ , provided *M* ≪ *N*. This reduction from cubic to a near-linear dependence on *N* (since *M* is fixed and small) is what enables the application of GPs to large-scale problems.

The quality of the SGP approximation is critically dependent on the number and the locations of the inducing points, **Z**. The number of inducing points introduces an inherent trade-off between modeling complexity (expressiveness) and computational efficiency. The number of inducing points, *M*, directly controls this balance. A small *M* provides the maximum computational speedup (minimal $O\left(M^{3}\right)$ cost) but limits the model’s capacity to capture complex local features while a large *M* is able to take in more complexity, but approaches the training time of the full GP. In higher dimensions (*D*), the volume of the space grows exponentially. Finding a small set of *M* inducing points that effectively cover a high-dimensional space becomes exceptionally difficult. To maintain a low approximation error, *M* often has to scale aggressively with *D*, which quickly degrades the computational savings.^27^ Additionally, the optimal placement of **Z** is treated as a set of hyperparameters to be learned or selected. The initial placement of **Z** is crucial for successful optimization. A common and effective noniterative approach is to use *K*-means clustering on the input training data **X**. By setting the *M* inducing points to the *M* cluster centroids derived from *K*-means, the inducing set is positioned to naturally reflect the high-density areas and the overall geometrical structure of the input space. This provides a robust, data-driven starting point that often accelerates subsequent optimization.^12^

For the final, refined placement, iterative methods based on gradient are typically used. One of the originally proposed methods is to use the marginal likelihood and maximize it using the inducing points on the full data set.^26^ An additional popular method is variational inference with the Evidence Lower Bound Objective (ELBO). This involves treating the inducing point locations **Z** as free parameters within a variational inference framework. The goal is to maximize the ELBO, which serves as a differentiable lower bound on the marginal likelihood of the data. Maximizing the ELBO not only optimizes the standard kernel hyperparameters but also drives the inducing points **Z** to locations that best approximate the true function posterior.^25,28^

The largest downside to SGPs is the loss of exactness. Because the inducing points are not actual data points from the data set, overgeneralizations can occur. As for the fine-tuning of the inducing points, there can be difficulties in the optimization. Depending on the number of inducing points, the optimization of the objective function can become high-dimensional (number of inducing points times inputs) and the optimization can be nonconvex. Also, during this optimization and inducing point selection, the uncertainty can deviate significantly from the full GP.^28,29^

### 2.3 Localized Gaussian Processes

While the SGP framework addresses the computational bottleneck of exact GPs by compressing the input space via inducing points, an alternative strategy is to partition the input space and model each region independently. This approach leads to localized Gaussian processes (LGPs). Instead of training a single, large GP over the entire data set (**X**, **y**), the input space **X** is divided into M localized regions. Each region *k* is associated with a small subset of the training data $D_{k}$ and is modeled by its own individual Gaussian process, GP_*k*_, often referred to as a local expert.^9,13,23,30,31^

This strategy effectively decouples the global data set size *N* from the complexity of any single model. By ensuring that the number of data points assigned to any local expert remains small (∑*N*_*k*_ = *N*, but *N*_*k*_ ≪ *N*), the computational cost of inverting the covariance matrix for each local model is drastically reduced.

The performance of LGP hinges on the mechanism used to define and allocate data to these local regions. The localization process often relies on a distance-based measure, *w*_*k*_(**x**), which determines the degree of activation or relevance of the *k*-th local expert for a given input **x**. Popular choices include *k*-means clustering and Euclidean distance for training and predictions.

Prediction at a query point x_*_ is performed by combining the outputs of the local experts, typically a set of $M_{\mathrm{active}}$ models in the neighborhood of x_*_. The final prediction, *y̅*(x_*_), is computed as a weighted average of the local mean predictions, *m*_*k*_(x_*_), where the weights are determined by the localization distance *w*_*k*_, as stated in eq 5 ^13^

5 $$\mathrm{y̅}\left(x_{*}\right)=\frac{\sum_{k=1}^{M_{\mathrm{active}}}w_{k}\left(x_{*}\right)m_{k}\left(x_{*}\right)}{\sum_{k=1}^{M_{\mathrm{active}}}w_{k}\left(x_{*}\right)}$$

where *m*_*k*_(x_*_) is the mean prediction made by the *k*-th local GP expert. Alternatively, the single closest expert can be selected. The choice of LGPs for prediction is a hyperparameter specific to the process.^13^

Within the domain of a specific local expert, the approximation error is incredibly low because it behaves exactly like a full GP for that localized data patch. It easily captures high-frequency, local details. However, the error can spike significantly at the boundaries between experts, leading to discontinuities or jagged predictions where the localized models try to hand off to one another. Additionally, the generalizability of the whole process can be missed, ignoring long-range global correlations that are present in the data. Localized methods rely entirely on spatial proximity. They need data points to be near each other to form a meaningful local neighborhood. In high dimensions, data points become inherently equidistant and isolated (the space is mostly empty). Defining meaningful “local patches” can break down completely in high dimensions without structural assumptions, causing localized models to overfit.^32^ Lastly, localized GPs are highly dependent on the partitioning of the data into each localized subset. If one subset contains significantly more data than the others, the computational training complexity can be near full GP complexity. Conversely, any partition with very little data, as in situations where outliers may be present, can result in particular localized GPs with poor local generalizability due to lack of data.

### 2.4 Decomposed Gaussian Processes

An alternative way of addressing the issue of training complexity is to instead approximate the Gaussian process as a series of basis functions. Reich et al. proposed the Bayesian Smoothing Spline-ANOVA (BSS-ANOVA) kernel, which uses Bernoulli polynomials to construct covariance terms for primary effects and interactions between inputs, along with scaling hyperparameters to produce the full kernel.^14^ If all input features are normalized to a [0,1] interval, then a single set of basis functions ϕ_*k*_ can be computed from the kernel using Karhunen-Loève decomposition.

The final decomposed BSS-ANOVA GP results in eq 6

6 $$f\left(x;\beta \right)=\beta_{0}+\sum_{i=1}^{d}\sum_{k=1}^{\infty }\beta_{\mathrm{ik}}\phi_{k}\left(x_{i}\right)+\sum_{i=1}^{d-1}\sum_{j=i+1}^{d}\sum_{k=1}^{\infty }\sum_{l=1}^{\infty }\beta_{\mathrm{ikjl}}\phi_{k}\left(x_{i}\right)\phi_{l}\left(x_{j}\right)+\cdot \cdot \cdot$$

where **β** are the weight distributions for each of the basis function terms (ϕ) and are assumed to be independent and identically distributed normal random variables.

This derivation can be generalized to other kernels and allows the Gaussian process to be approximated by including a limited number of basis function terms, creating a linear Bayesian equation that can be solved in $O\left({\mathrm{NP}}^{2}\right)$ via ordinary least-squares, which is faster than traditional Gaussian process training of $O\left(N^{3}\right)$ . Two of the common issues that appear in basis function approaches are the choice of basis functions and the number to include. This is addressed as the eigenvalue decomposition orders the ϕ basis functions by complexity, (similar to Principle Component Analysis which is a specific case of Karhunen-Loève decomposition) enabling a logical addition of terms. To determine the number of terms used, this methodology uses a forward variable selection where the model is evaluated on both accuracy and complexity using the Bayesian Information Criterion to determine the correct number of basis functions to include. This framework is formally known as FoKL-GPs (Forward variable selection using Karhunen-Loève decomposed Gaussian Processes).^14,15^

Because this model now takes the form of a set of basis functions, traditional Bayesian model building advantages can be applied. One such advantage is that the distributions of the **β** parameters that allow the training data to be reconstructed from the model. In other words, the model stores information about the training data within its parameters. This means that incremental training of the model only requires presenting new data points and the previously trained model’s **β** distributions, leading to faster training times that depend only on the number of new data points added to the training set. To be compatible with the forward variable selection in cases of updating a previously trained model with new data, cases for a model with the same number of terms as the previous model and more terms need to be taken into account. These developments are novel, but are outside the core scope of this paper and are included in the Supporting Information.

One of the downsides to this approach is that because the decomposition of the kernel happens for a normalized set of inputs, the resulting basis functions are only defined on the interval [0,1]. This means that the bounds of the inputs of the model need to be defined before the initial training and after the model is trained, it cannot extrapolate past preset input ranges. Additionally, the error depends entirely on whether the chosen decomposition matches the physical reality of the system. If the true function is highly additive or mostly driven by main effects and low-order interactions, the approximation error is remarkably low. However, if the system relies on complex, high-order cooperative interactions that the selected decomposition omits, those effects are lost, increasing the structural error. Decomposed GPs are explicitly designed to combat the curse of dimensionality. By restricting the kernel interactions to lower-dimensional manifolds, the model bypasses the need to learn a massive *D*-dimensional covariance matrix all at once. This makes them highly robust in high-dimensional spaces, provided the underlying system is not completely coupled across all dimensions simultaneously.^33^

### 2.5 Model Predictive Control

Model Predictive Control (MPC) is a control strategy that uses a model of the process to predict future behavior and optimize control actions over a finite time horizon. At each sampling instant, the controller solves an optimization problem to minimize a cost function, typically involving the deviation of the predicted output from a reference trajectory or set point, subject to constraints on inputs, outputs, and states. Only the first element of the optimal control sequence is applied to the plant, and the process is repeated at the next time step with updated state information.^34–37^

Formally, at each time step *k*, the MPC solves an optimization problem to determine a sequence of control moves over a control horizon *N*_*c*_, based on process predictions over a longer prediction horizon *N*_*p*_. The objective is usually to minimize a quadratic cost function *J* that balances set point tracking and control effort, often represented as shown in eq 7

7 $$J=\sum_{i=1}^{N_{p}}∥\mathrm{x̂}\left(k+i|k\right)-x_{\mathrm{ref}}\left(k+i\right){∥}_{Q}^{2}+\sum_{j=0}^{N_{c}-1}∥u\left(k+j|k\right){∥}_{R}^{2}$$

where *x̂*(*k* + *i*|*k*) is the predicted state vector at step *k* + *i*, *x*_ref_ is the reference set point, *u* is the control input vector, and **Q** and **R** are weighting matrices. This minimization is performed subject to constraints on relevant quantities such as inputs, outputs, and states.

The performance of the MPC relies heavily on the accuracy of the internal process model. In many industrial applications, the process dynamics may change over time due to fouling, catalyst deactivation, equipment wear, or changes in operating conditions. When the mismatch between the model and the true plant becomes significant, the controller performance degrades, potentially leading to constraint violations or instability. To maintain optimal performance, it is necessary to detect this degradation and update the model, a process often referred to as model re-identification.^11,16–18^

### 2.6 Re-identification Metrics

A critical design choice for online learning MPC systems is the selection of the evaluation metric that triggers model re-identification. Continuously retraining the model can be computationally expensive and may lead to overfitting if the new data is not informative. Therefore, a key challenge in re-identification is determining when to trigger the re-identification process. This decision is typically based on monitoring a performance metric or a model quality index. Multiple theoretically motivated approaches exist that significantly influence controller performance.^11,16–18^ Four candidate re-identification triggers are used in this work.

#### 2.6.1 Prediction Uncertainty

Gaussian processes inherently provide a posterior variance that quantifies prediction uncertainty in addition to the mean prediction used in the deterministic GP-MPC implementation. This uncertainty is minimal near training data and increases in regions of sparse data, reflecting epistemic uncertainty regarding unobserved input space regions.^19^ The underlying hypothesis is that retraining should be triggered when Gaussian process uncertainty exceeds a threshold, signaling that the system is operating in a poorly characterized region of the input space. Under this logic, retraining during exploration of new input regions should yield improved model predictions in those regions.

#### 2.6.2 Maximum Mean Discrepancy Covariate Shift

An alternative hypothesis proposes monitoring the input space distribution directly to detect shifts into previously unobserved regions. Kernel-based methods that leverage Reproducing Kernel Hilbert Space (RKHS) structure provide a principled approach to this problem.^38,39^ Maximum Mean Discrepancy (MMD) is a nonparametric metric that quantifies the distributional distance between historical training data and current operational data.^40^

The MMD is defined as the maximum difference between the expectations of any function *f* (with unit norm) drawn from the two distributions. Using the kernel trick, the squared MMD (MMD^2^) can be expressed using the expectations of the kernel function as shown in eq 8

8 $${\mathrm{MMD}}^{2}\left(H,P,Q\right)=E_{x,x\prime ∼P}\left[k\left(x,x^{\prime }\right)\right]+E_{y,y\prime ∼Q}\left[k\left(y,y^{\prime }\right)\right]-2E_{x∼P,y∼Q}\left[k\left(x,y\right)\right]$$

where *x*, *x*′ are independent samples from *P* (Source), and *y*, *y*′ are independent samples from *Q* (Target). The unbiased empirical estimator (U-statistic) for the squared MMD, which matches the implementation logic, is used to estimate MMD^2^ from *n* source samples (*x*_*i*_) and *m* target samples (*y*_*j*_) as shown in eq 9.^41^

9 $${\mathrm{MMD}}_{U}^{2}=\underset{\mathrm{Source}‐\mathrm{Source}\mathrm{Similarity}}{\underset{︸}{\frac{1}{n\left(n-1\right)}\sum_{i\neq j}^{n}k\left(x_{i}\;,x_{j}\;\right)}}+\underset{\mathrm{Target}‐\mathrm{Target}\mathrm{Similarity}}{\underset{︸}{\frac{1}{m\left(m-1\right)}\sum_{i\neq j}^{m}k\left(y_{i}\;,y_{j}\;\right)}}-\underset{\mathrm{Source}‐\mathrm{Target}\mathrm{Dissimilarity}}{\underset{︸}{\frac{2}{\mathrm{nm}}\sum_{i=1}^{n}\sum_{j=1}^{m}k\left(x_{i}\;,y_{j}\;\right)}}$$

#### 2.6.3 ITAE

The Integral of Time-weighted Absolute Error (ITAE) is a widely used performance index in control system design to evaluate and optimize the transient response of a control loop. The core idea behind the ITAE metric is to evaluate the error between the set point and the process variable. If an error persists, such as an offset, the error will accumulate. This can be used in conjunction with a threshold value for ITAE in order to determine when the model should be retrained.^42^ The ITAE is defined mathematically in eq 10

10 $$\mathrm{ITAE}=\int_{0}^{\infty }t\cdot \left|e\left(t\right)\right|dt$$

where *e*(*t*) is the error (difference between the set point and the controlled variable) and *t* is time.

ITAE is favored for its unique characteristic of penalizing errors that persist over time more heavily than errors that occur initially. The emphasis on errors later in the response encourages a faster and smoother response, typically results in a closed-loop response with a good balance and often yielding low overshoot and a rapid decay of oscillations to achieve the steady state (zero error).

#### 2.6.4 MP-ITAE

Model Prediction-ITAE (MP-ITAE) offers an alternative metric that evaluates model accuracy rather than control performance. Whereas ITAE measures the fidelity of closed-loop tracking, MP-ITAE compares model predictions directly against measured values, assessing model quality independent of control performance. This methodology operates on the premise that accurate model predictions enable effective MPC control. For this system, MP-ITAE compares the model-predicted dynamics against finite difference approximations of the measured height derivatives as shown in eq 11

11 $$\mathrm{MP}‐\mathrm{ITAE}=\int_{0}^{\infty }t\cdot \left|{\hat{\overset{\;}{h}}}_{i}\left(t\right)-{ḣ}_{i}\left(t\right)\right|dt=\sum_{0}^{\infty }t\cdot \left|\delta_{i}\left(t-\Delta t\right)-\frac{h_{i}\left(t\right)-h_{i}\left(t-\Delta t\right)}{\Delta t}\right|\Delta t$$

where δ is the GP prediction and Δ*t* is the time step.

### 2.7 Stability and Recursive Feasibility

Stability guarantees are critical when assessing the performance of model predictive control systems, particularly for nonlinear processes. While linear MPC systems benefit from well-established stability conditions, nonlinear systems present significant theoretical challenges. The inherent nonlinearity of the process dynamics, coupled with the use of data-driven surrogate models like Gaussian processes, complicates the direct application of classical stability analysis. Two foundational pillars of MPC theory address these challenges. Local stability analysis via linearization and eigenvalue computation provides stability guarantees, while recursive feasibility analysis through terminal constraint design ensures the MPC optimization remains solvable throughout operation.

#### 2.7.1 Linearization and Eigenvalue-Based Stability Analysis

For a general nonlinear discrete-time system described by **x**(*k* + 1) = *f*(**x**(*k*), **u**(*k*)), where $x\in R^{n}$ is the state vector, u∈R is the control input, and *k* denotes the time step, direct stability analysis is often intractable. A standard approach is to linearize the dynamics around an equilibrium point (x*, u*), where x* = *f*(x*, u*). The linearized system is characterized by the Jacobian matrices as shown in eq 12.

12 $$A_{d}={\frac{\partial f}{\partial x}|}_{\left(x*,u*\right)},\;B_{d}={\frac{\partial f}{\partial u}|}_{\left(x*,u*\right)}$$

The dynamics of small deviations δ**x**(*k*) = **x**(*k*) – x* and δ**u**(*k*) = **u**(*k*) – u* from equilibrium are approximated in eq 13 by the linear system.

13 $$\delta x\left(k+1\right)=A_{d}\delta x\left(k\right)+B_{d}\delta u\left(k\right)$$

For controller design, a linear feedback law δ**u**(*k*) = −**K**δ**x**(*k*) is typically used, where **K** is the feedback gain matrix. Lyapunov’s linearization theorem provides the fundamental stability criterion that if all eigenvalues of the closed-loop system matrix (**A** *_d_* – **B** *_d_* **K**) have magnitude strictly less than one, then the equilibrium point is locally asymptotically stable.^43–45^ The gain matrix **K** can be obtained through various controller design methods.

For first-principles models, the Jacobian matrices **A** *_d_* and **B** *_d_* can often be computed analytically, while for data-driven models such as Gaussian processes, the Jacobians may be calculated analytically or approximated using finite differences depending on the model structure. This approach enables stability analysis of surrogate models by computing their local linear behavior at equilibrium points. When GP models are updated online with new data, tracking eigenvalue changes provides insight into whether retraining improves or degrades the model’s representation of system dynamics.^46^

While linearization provides valuable local insights, its primary limitation is that stability guarantees hold only in a neighborhood of the equilibrium. The method also does not address constraint satisfaction, which is critical in practical MPC implementations where state and input bounds must be respected.

#### 2.7.2 Recursive Feasibility and Terminal Constraints

A distinct but equally important theoretical concern for MPC is recursive feasibility. This is ensuring that if the optimization problem is feasible at time *k*, it remains feasible at all subsequent time steps. Standard finite-horizon MPC formulations, such as the one presented in eq 7, do not inherently guarantee this property. Without recursive feasibility, the controller may unexpectedly fail to find a solution during operation, leading to safety concerns or process upsets.

A classical solution to this problem is the introduction of a terminal constraint in the MPC formulation as shown in eq 14 ^45,47^

14 $$x\left(k+N_{p}\right)\in \Omega_{f}$$

where Ω_*f*_ is a positively invariant set under a terminal control law κ­(**x**). eq 15 defines the condition for positive invariance for the terminal set Ω_*f*_.

15 $$x\in \Omega_{f}\Rightarrow f\left(x,\kappa \left(x\right)\right)\in \Omega_{f}$$

This property ensures that once the state enters the terminal set, it can be maintained within the set indefinitely using the terminal controller κ­(**x**). At each time step, the MPC optimization includes the constraint that the predicted terminal state must lie within Ω_*f*_. The recursive feasibility proof states that if a feasible control sequence exists at time *k*, then at time *k* + 1, a candidate feasible trajectory can be constructed by appending the terminal controller action to the remaining control sequence from the previous solution. Because the terminal state was in Ω_*f*_ and Ω_*f*_ is positively invariant, feasibility is preserved.

A common approach to constructing terminal sets for linear or linearized systems is to use ellipsoidal sets defined by the solution to the Discrete-Time Algebraic Riccati Equation (DARE). For a discretized linear system **x**(*k* + 1) = **A**_*d*_ **x**(*k*) + **B**_*d*_ **u**(*k*) and cost matrices **Q** and **R**, the DARE is solved for the positive definite matrix **P**, as shown in eq 16.

16 $$P=A_{d}^{T}{\mathrm{PA}}_{d}-A_{d}^{T}{\mathrm{PB}}_{d}{\left(R+B_{d}^{T}{\mathrm{PB}}_{d}\right)}^{-1}B_{d}^{T}{\mathrm{PA}}_{d}+Q$$

The optimal linear quadratic regulator (LQR) gain is then given by eq 17.

17 $$K={\left(R+B_{d}^{T}{\mathrm{PB}}_{d}\right)}^{-1}B_{d}^{T}{\mathrm{PA}}_{d}$$

Here, the terminal controller is κ­(**x**) = – **K**(**x** – x*) + u*. The corresponding terminal set is an ellipsoid centered at the equilibrium, as expressed in eq 18:

18 $$\Omega_{f}=\left\{x\in R^{n}:{\left(x-x^{*}\right)}^{T}P\left(x-x^{*}\right)\leq c\right\}$$

where the scalar parameter *c* > 0 determines the size of the set. The maximum feasible value of *c* is determined by ensuring that all state and input constraints are satisfied throughout Ω_*f*_ under the terminal control law.^45^

The linearization and terminal constraint approaches presented above provide practical tools for analyzing nominal stability and recursive feasibility of nonlinear GP-MPC systems. Alternative formulations like robust MPC methods (such as Tube-based MPC) can explicitly incorporate the probabilistic uncertainty estimates provided by Gaussian processes, tightening constraints based on prediction variance to provide stochastic guarantees on constraint satisfaction.^45^

## 3 Methodology

The effectiveness of various GP methodologies, including different kernel choices, training techniques, and integration with physical models, will be evaluated using a multistage approach. The cascaded tanks problem serves as the motivating example for this comparison, representing a standard control system benchmark in which the flow rate into a primary tank is regulated via a control valve, with the first tank then gravity-fed into the second.^48^ The system configuration is shown in Figure 1. The second tank exhibits nonlinear height dynamics with respect to changes in control action and tank heights, establishing a nonlinear control system for GP modeling. The evaluation framework consists of the following phases.

1. Offline open-loop verification: This phase performs offline data analysis to establish baseline performance for different GP models and identifies suitable kernel functions and methodologies capable of accurately capturing the inherent nonlinear dynamics of the cascaded tanks system.
2. Closed-loop MPC validation: This phase uses an online simulation and an experimental routine covering both model extrapolation and interpolation to validate both MPC performance given system states and improvement in MPC performance with more data.
3. Evaluation of re-identification metrics: This phase uses online simulations of the cascaded tanks system to generate synthetic data and evaluate the efficacy of re-identification criteria that determine when model retraining is warranted.
4. Evaluation of GP algorithms: This phase continues with online simulations to examine the computational efficiency and adaptability of GP methodologies when data arrives sequentially.
5. Evaluation of Stability and Recursive Feasibility: This phase evaluates the chosen GP approximation and re-identification combination to assess stability and recursive feasibility through a multiset point analysis of the linearized system across the system’s operating ranges.
6. Experimental system validation: The final phase investigates hybrid modeling approaches that integrate system-specific physical knowledge by applying the controls to a real-world cascaded tank apparatus at the WVU Unit Operations Lab. This approach leverages benefits of physical knowledge, including interpretability, while using GPs to model additional unaccounted dynamics and nonidealities.

**Figure 1:**
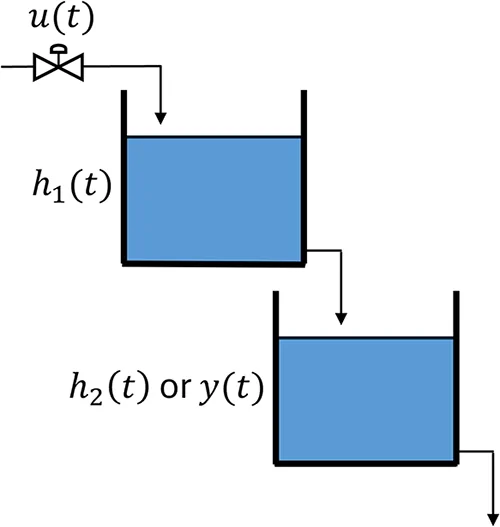
Cascaded tanks setup.

### 3.1 Offline Open-Loop Verification

Experimental tank height data from prior literature^48^ is used in this step. This is representative of having limited or only related data early on in design work where data fully characterizing the process is not available. However, if data is available from the specific process, that is preferential.

Gaussian process models are trained on tank height dynamics derived from finite difference approximations to assess kernel selection impacts on prediction accuracy. Derivative approximation via backward finite differences amplifies measurement noise variance by $\frac{2}{\Delta t^{2}}$ . For the 3-s sampling interval, the derivative noise standard deviation becomes $\frac{\sqrt{2}}{\Delta t}\sigma \approx 0.47\sigma$ , where σ is the measurement noise. The signal-to-noise ratio remains favorable as pressure transmitter noise is small relative to system dynamics during transient operation.

The data set is comprised of 12,500 s of measurements at 5 s intervals, partitioned into training (first 10,000 s) and validation (final 2500 s) sets using an 80/20 split. The system dynamics are modeled in eqs 19 and 20

19 $$\dot{h_{1}\;}=\delta_{1}\left(u,h_{1},h_{2}\right)$$

20 $$\dot{h_{2}\;}=\delta_{2}\left(u,h_{1},h_{2}\right)$$

where *u* is the control valve position, *h*_1_ is the height of tank 1, *h*_2_ is the height of tank 2.

### 3.2 Closed-Loop MPC Validation

Following the offline evaluation of the GP approximation techniques, their performance is evaluated in an online setting using a digital simulation of the cascaded tanks system, with dimensions taken from the apparatus in question, the physical system setup at the WVU Unit Operations Lab. The outlet flow rate from each tank is modeled according to Torricelli’s law, which characterizes the outlet flow rate as proportional to the square root of the fluid height. The system dynamics are described by a nonlinear state-space model with the fluid height in the second tank as the measured output. Measurement noise is incorporated to represent transmitter uncertainty, with an amplitude corresponding to three standard deviations of 0.10% of the full span (0–30 in.). The inlet flow rate to the first tank serves as the control input, while external disturbances are not explicitly modeled. The state-space model is expressed as shown in eqs 21, 22, and 23.

21 $$ẋ=\left[\begin{array}{l} {ḣ}_{1}\\ {ḣ}_{2} \end{array}\right]=\left[\begin{array}{l} \frac{q_{\mathrm{in}}}{A_{1}}-\frac{a_{1}\sqrt{2g}}{A_{1}}\sqrt{h_{1}}\\ \frac{a_{1}\sqrt{2g}}{A_{2}}\sqrt{h_{1}}-\frac{a_{2}\sqrt{2g}}{A_{2}}\sqrt{h_{2}} \end{array}\right]$$

22 $$y=h_{2}$$

23 $$q_{\mathrm{in}}=q_{\max}u$$

where *h*_1_ and *h*_2_ are the heights of the fluid in the first and second tanks, respectively, *q*_in_ is the inlet flow rate assuming a linear relationship with *u*, the control valve position defined as percent open, *A*_1_ and *A*_2_ are the cross-sectional areas of the tanks, *a*_1_ and *a*_2_ are the cross-sectional areas of the outlet pipes, *g* is the acceleration due to gravity, *y* is the output of the system, and *q*_max_ is the maximum flow rate of the control valve at 100% opening. The values of the system parameters can be found in Table 1. Figure 2 presents a complete MPC architecture incorporating the four proposed re-identification methods. For this step, only the initialization and normal operation blocks are considered. The details of the inputs and the outputs of the blocks in the MPC diagram are as follows.

1. Initial PID Training Data: Data is collected from the system/simulation of interest where a PID is used for controls. The output of this block is the measured states collected from the system.
2. Model Initialization: The inputs to this block are the states collected from the system/simulation under PID control. This block takes this data and creates an initial GP model of the dynamics of the system/simulation. The model objects created are the outputs of this block.
3. Model Predictive Control: This block contains multiple inputs, including the model objects either created from model initialization or model retraining, the current set point of the system, and the current states of the system/simulation. This block takes the model of the dynamics and the states provided by the system in order to solve the initial value problem of predicting the states of the system at future timesteps. An optimization is ran to determine the optimal control inputs to minimize the difference between the set point and the state of the system at each time step as described in eq 7. The output of this block is the optimal control policy of the next step only and any states needed for the re-identification criterion selected.
4. Cascaded Tanks System/Simulation: This block takes the optimal control input for the next time step and applies it to the system or runs the simulation one time step forward using the optimal control input. The output of this block is the states of the system.
5. Re-identification Criterion: The input to this block is dependent on the necessary information on the re-identification metric of choice (model prediction uncertainty, historical states, or model predictions). The re-identification metric is then calculated. If the value calculated passes a threshold, this block’s output is a boolean value of True. Otherwise, the output is a boolean value of False.
6. Model Retraining: The input to this block is a boolean value of True or False. If it is True, the initial PID training data and the historical data collected from the system/simulation will be used to retrain the models of the dynamics of the system. The model objects created are the outputs of this block.

**Figure 2:**
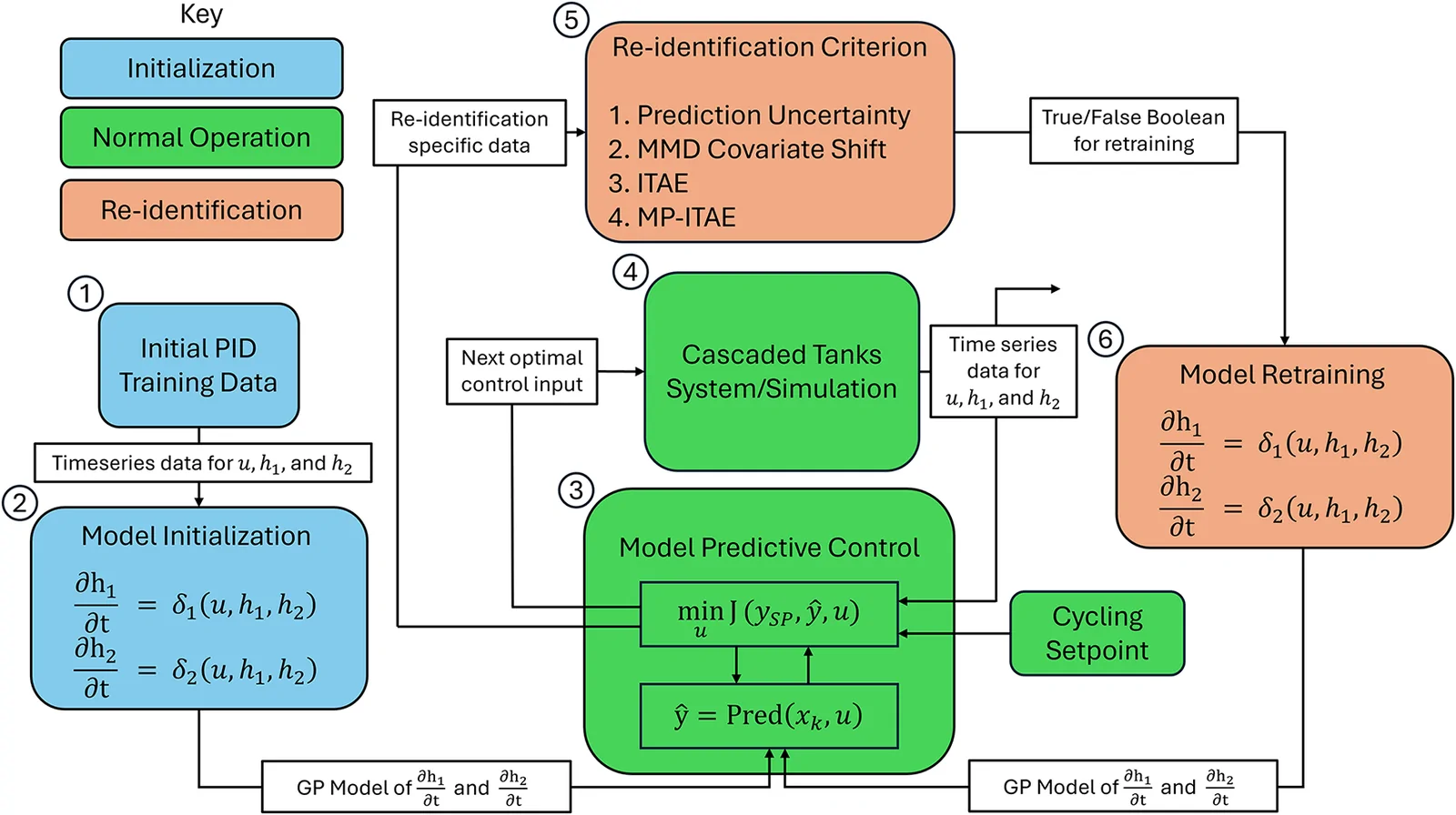
MPC diagram with re-identification.

**Table 1:** System Parameters

Parameter	Value
*a*_1_	0.10 in^2^
*a*_2_	0.04 in^2^
*A*_1_	11.79 in^2^
*A*_2_	11.79 in^2^
*g*	$386.1\;\frac{\mathrm{in}}{s^{2}}$
*q*_max_	$6.77\;\frac{{\mathrm{in}}^{3}}{s}$

A warm start SLSQP (Sequential Least Squares Programming) optimizer is used to solve the MPC optimization with a forward Euler’s method to integrate the ODE system. For training the MPC controller, it is assumed that historical PID control data is available, as, in practice, MPC implementations typically come after simpler control strategies are used and do not provide sufficient control. To assess the generalizability of the MPC controller and validate the benefit of increased training data, a systematic training protocol was developed. The initial training data set is generated by simulating the system under PID control at two set points for the height of the second tank (10 and 20 in.) for 600 s, providing a realistic baseline data set characterized by high volume and low variance. For validation, the MPC controller is evaluated across five set points (10, 20, 25, 15, and 5 in., in that order) to assess both interpolation and extrapolation capabilities. This validation protocol is repeated using additional set points of PID training data to validate the effectiveness of retraining the MPC.

### 3.3 Evaluation of Re-identification Metrics

The re-identification triggers for this work are organized into two distinct categories. The first category assumes retraining is necessary when the system explores new regions of the input space. This hypothesis is tested through two approaches: (1) Gaussian process prediction uncertainty, which assumes high uncertainty correlates with unexplored input regions, and (2) Maximum Mean Discrepancy (MMD) covariate shift, which quantifies distributional changes between historical training data and current operational data. The second category evaluates model quality directly through control performance metrics: (1) Integral Time-weighted Absolute Error (ITAE), a classical controls metric that penalizes persistent tracking errors, and (2) Model Prediction-ITAE (MP-ITAE), which evaluates model accuracy by comparing predicted and observed system dynamics. To identify an acceptable threshold for each metric, a Pareto curve can be generated between the performance of the model (e.g., MSE or tracking error) and computational cost (e.g., total training time, number of retraining events). The choice of threshold is a qualitative decision based on the dynamics of the particular system and available computational resources.

### 3.4 Evaluation of GP Algorithms

After the validation of the MPC system and choice of re-identification metrics have been selected, the focus shifts to evaluating Gaussian process approximation techniques and comparing their trade-offs in computational efficiency and prediction accuracy. The candidate methods include one standard GP regression method and two methods from each of the GP approximation categories (sparse GPs, localized GPs, and decomposed GPs) based on the offline open-loop validation results.

### 3.5 Evaluation of Stability and Recursive Feasibility

A multiset point evaluation is performed across the cascaded tanks system’s operating range. For the cascaded tanks system, which operates over a wide range of set points (5–25 in.), the linearized system matrices and corresponding stability characteristics may vary significantly with the operating point.

Eigenvalue-based stability analysis and terminal set design must be performed at multiple representative set points throughout the operating range. At each set point, equilibrium points are computed from the steady-state balance equations, the system is linearized to obtain Jacobian matrices **A** and **B**, and the DARE is solved to obtain both the LQR gain matrix **K** (for eigenvalue analysis) and the matrix **P** (for terminal set construction). The eigenvalue spectra of the closed-loop system (**A** – **BK**) quantify local stability margins, while the maximum feasible terminal set scaling parameter *c* indicates constraint tightness at each operating point.

For the deterministic GP-MPC implementation, this multiset point analysis serves the additional critical purpose of model validation. eigenvalue spectra are computed for three models at each set point: the first-principles physical plant model, the initial GP model trained on PID data, and the final GP model after online updates. Close agreement between GP and physical model eigenvalues across the operating range validates that the data-driven surrogate accurately captures the essential dynamic behavior of the true system. Significant discrepancies at specific operating points identify regions where additional training data or model refinement may be necessary to ensure reliable controller performance.

### 3.6 Experimental System Validation

This section presents the application of the MPC framework selected to a real-world cascaded tanks experiment at the WVU Unit Operations Lab. Real-world systems introduce numerous complexities absent from simulation, including nonlinearities in the control valve, time delays in control action, hydrodynamic delays between tanks, siphoning phenomena, and other system nonidealities.

To improve the MPC at this stage and deal with any accuracy loss accrued by approximating the GPs, additional physics can be added to create hybrid models in order to assess whether theoretical assumptions generalize to the practical implementation. Hybrid machine learning approaches encode known physical principles into the model structure, reducing the complexity of phenomena the machine learning must capture and thereby enhancing accuracy, generalizability, and training time.^1,49–52^ While several methods are available in literature, two methodologies were chosen for this work due to the speed of their trainings: feature engineering and residual modeling.

Feature engineering transforms the input features to better capture known physical phenomena.^49,52^ In this case, Torricelli’s law is applied, which states that the outlet flow rate from each tank is proportional to the square root of the tank height. This relationship is incorporated through the modified input features shown in eqs 24 and 25.

24 $$\dot{h_{1}}=\delta_{1}\left(u,\sqrt{h_{1}},\sqrt{h_{2}}\right)$$

25 $$\dot{h_{2}}=\delta_{2}\left(u,\sqrt{h_{1}},\sqrt{h_{2}}\right)$$

The second method is residual modeling, which combines a first-principles model with machine learning to predict the residual between the measured system dynamics and the first-principles prediction of the dynamics. This residual captures phenomena not accounted for by the physics-based model.^1,53^ For this application, Torricelli’s law forms the basis of the first-principles model, with the dynamics represented in eq 26.

26 $$\left[\begin{array}{l} {\hat{ḣ}}_{1}\\ {\hat{ḣ}}_{2} \end{array}\right]=\left[\begin{array}{l} k_{1}u^{\alpha }-k_{2}\sqrt{h_{1}}\\ k_{2}\sqrt{h_{1}}-k_{3}\sqrt{h_{2}} \end{array}\right]$$

An optimizer is used with initial PID data to calculate parameters *k*_1_, *k*_2_, *k*_3_, and exponent α. The control valve signal is raised to the power α to better capture control valve nonlinearities. Due to the absence of a valve characteristic curve for the experimental setup, the relationship between valve position and actual flow rate must be identified empirically as part of the system model. The Gaussian process then learns the residual between the observed data and the physics-based predictions, as shown in eqs 27 and 28.

27 $$\dot{h_{1}}-{\hat{ḣ}}_{1}=\delta_{1}\left(u,h_{1},h_{2}\right)$$

28 $$\dot{h_{2}}-{\hat{ḣ}}_{2}=\delta_{2}\left(u,h_{1},h_{2}\right)$$

## 4 Results and Discussion

This section details the results of the proposed framework as applied to a cascaded tanks system.

### 4.1 Offline Open-Loop Verification

Four kernels (exponential, Matern, squared exponential, and Bernoulli) were evaluated using an initial value problem framework, given the system’s initial state and control valve trajectory specified over the entire data set. Performance was quantified using mean squared error (MSE) between measured and predicted height of the second tank on the last 20% of the timeseries data. All simulations were performed on standard desktop-level hardware (32 GB RAM, 3.4 GHz Processor). The results are presented in Table 2 and Figure 3.

**Figure 3:**
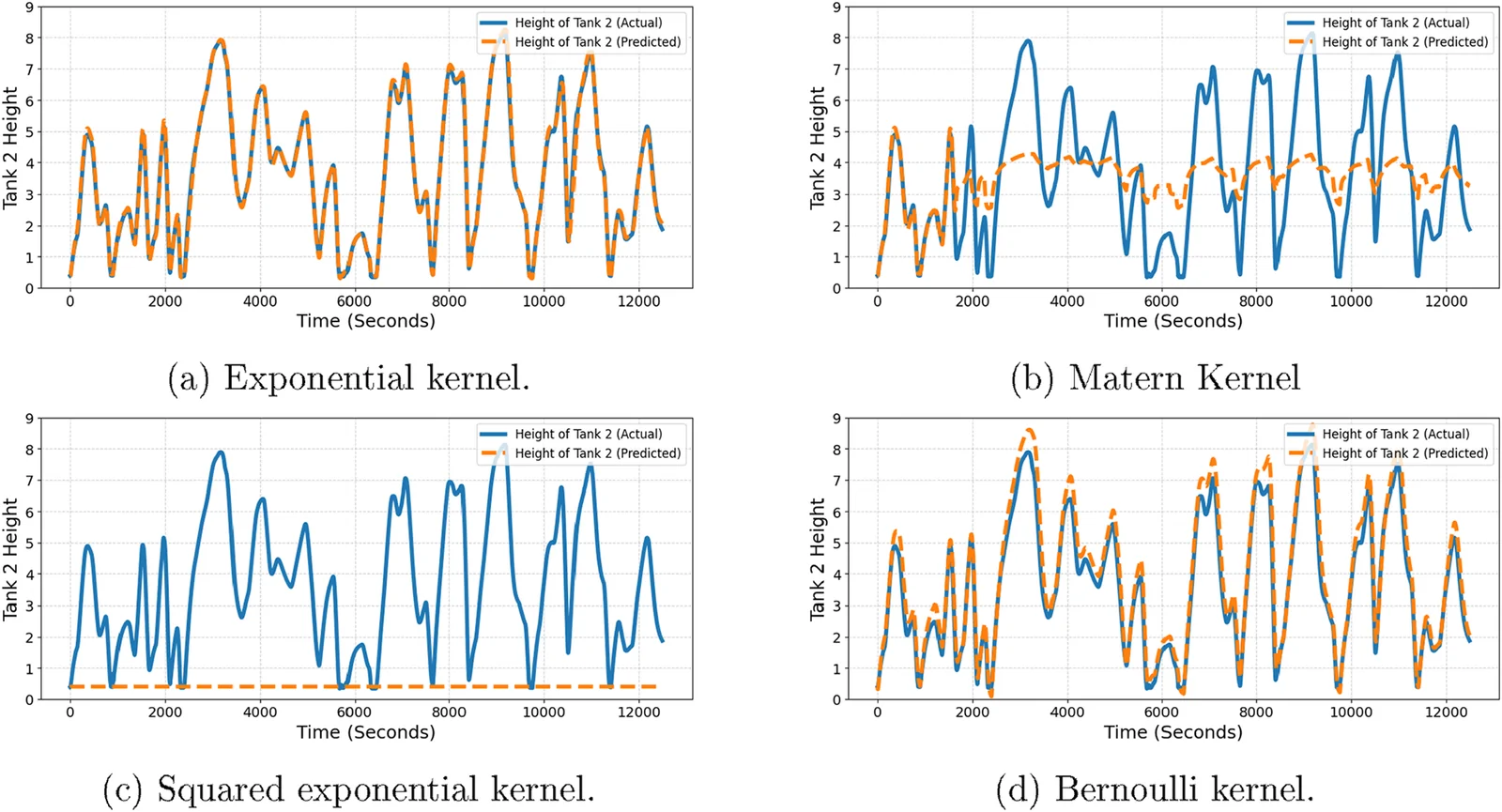
Comparison of four different kernels on offline data.

**Table 2:** A Table Comparing Kernels and MSE

Kernel	Mean Squared Error (MSE)	Training Time (s)
Exponential	0.0087	23.9
Matern (ν = 1.5)	0.5374	28.7
Squared Exponential	3.2297	5.7
Bernoulli	0.0346	12.1

Comparative analysis indicates that the exponential kernel was the best kernel function to captured the observed system dynamics, which is consistent with the expected nonsmooth characteristics of tank-height derivatives. Validation on the withheld test set (final 20%) confirmed the model’s ability to generalize, with no evidence of overfitting. The Matern kernel (ν = 1.5) produced acceptable predictions for roughly the first 1,500 s, after which its responses became excessively attenuated and failed to capture large transients. The squared exponential kernel was unable to represent the dynamics at all, indicating that its smoothness assumptions are incompatible with the observed behavior. In this case the trained model regressed to the uninformative prior (δ = 0), effectively predicting negligible dynamics independent of valve position. The Bernoulli kernel captured most trends but had errors during rapid state changes. On the basis of these results, the exponential kernel was selected for all subsequent GP evaluations.

With the kernel choice fixed, strategies to improve computational efficiency were evaluated. Three approaches were considered: SGPs (inducing points), localized GPs, and decomposed GPs. For the SGP method, three strategies were used to identify inducing points: *K*-means clustering alone, Maximum Marginal Likelihood (MML) with *k*-means initialization, and Evidence Lower Bound Objective (ELBO) with *k*-means initialization. All methods reduced the training data set from 2,000 to 100 points (5% of original size). Iterative optimization methods executed 500 training iterations. Results are presented in Table 3.

**Table 3:** A Table Comparing SGP Methods and MSE

Method	MSE	Training Time (s)
*K*-Means Only	0.0131	1.6
ELBO	0.0100	23.6
Maximum Marginal Likelihood	0.0061	13.7

All three methods successfully captured system dynamics. *K*-means clustering performed least favorably due to lack of optimization of inducing point locations, yet achieved the fastest training time by a significant margin. The ELBO method improved prediction accuracy but required substantially longer training. The MML method demonstrated superior performance, achieving lower error than the full GP. This result is possible because SGP inducing points represent optimally placed artificial points rather than actual data points. Compared to full GP training with the exponential kernel, MML reduced training time by approximately 50%. However, this is not as large of an improvement from the theoretical computational advantage as the algorithm is required to solve the $O\left({\mathrm{NM}}^{2}\right)$ training objective multiple times (500 iterations here). Although MML produced error less than half that of *k*-means, it required approximately 8.5 times longer for iterative optimization to complete. These results demonstrate that sparse GPs represent a viable option for accelerating training on this system.

The localized GP method was subsequently evaluated. Local GP clusters were defined via *K*-means clustering, with predictions generated by activating only the nearest local expert, identified by proximity to the *K*-means centroid. Results are presented in Table 4.

**Table 4:** Localized Methods Comparison

Number of Local GPs	MSE	Training Time (s)
2	0.0091	8.0
3	0.0094	2.7
4	0.0105	2.0
5	0.0093	2.8
6	0.0079	1.1
7	0.0076	1.7
8	0.0173	0.8
9	0.0121	0.7

The localized GP method successfully captured system dynamics with substantially reduced training time. All used only the single closest expert to make predictions. The best performing configuration used seven local GPs with a training time of 1.7 s. These results establish the localized GP method as a viable option for accelerating training on this system. One observation is that training time reduction did not exhibit a continuous decrease but rather a stepwise pattern with a level of randomness. Significant training acceleration occurs only when adding a local GP partitions the largest preceding cluster, the primary training bottleneck. Such transitions appear at 2–3, 5–6, and 7–8 local GPs. However, there is no guarantee that the number of data points in the largest local expert goes down with an increase in the number of total local GPs, leading to occasional increases in training times with an increasing number of local GPs. Additionally, increasing the number of local GPs elevates overfitting risk, increasing error beyond approximately seven local experts.

The final evaluations examined the decomposed GP approximations using basis sets derived from the two highest-performing kernels in the initial evaluation, the exponential and the BSS-ANOVA (Bernoulli based) kernels. Both approaches used forward variable selection to determine the number of basis functions to include. Results are presented in Table 5 and Figure 4. Training times for the decomposed GP methodologies include both the forward variable selection for the model buildout and the Bayesian sampling of the final model parameters.

**Figure 4:**
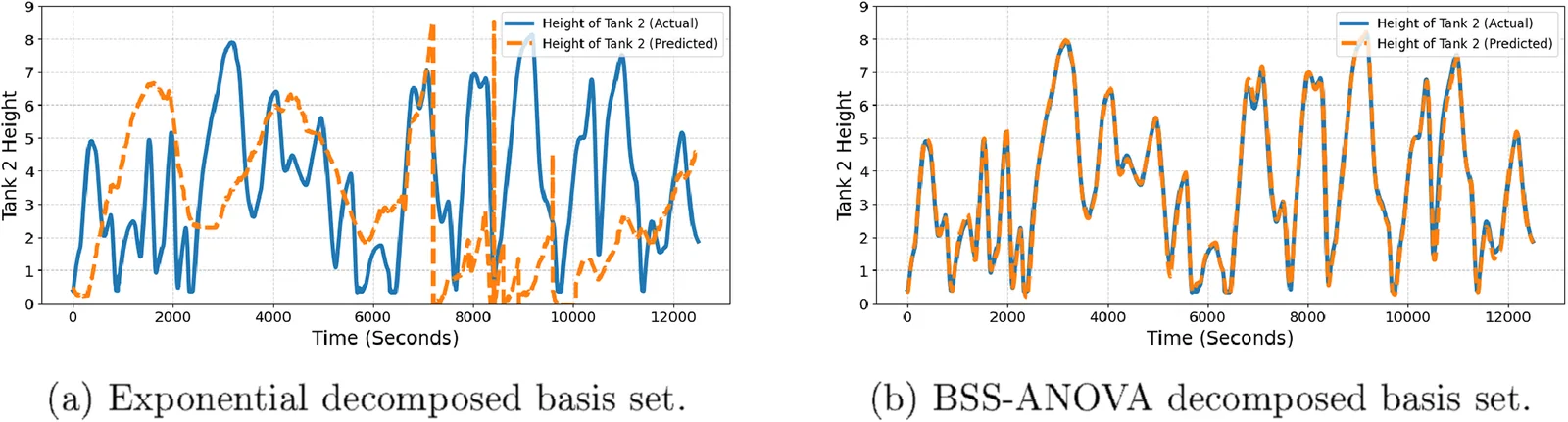
Decomposed GP method result.

**Table 5:** Decomposed GP Methods Comparison

Method	MSE	Training Time (s)
Exponential	0.5037	3.7
BSS-ANOVA	0.0118	7.7

The exponential decomposed basis set failed to predict system dynamics accurately, whereas the BSS-ANOVA basis set demonstrated successful performance. This discrepancy reflects the fact that decomposed basis set expressiveness does not directly correlate with kernel performance in the full GP context. The differing numbers of basis function terms between methods, evidenced by the exponential model’s substantially faster training time, further explains this performance divergence. Although the BSS-ANOVA method accurately captured system dynamics, it exhibited higher error compared to other GP approximation methods.

The decomposed GP method uses ordinary least-squares to solve for architecture, achieving a substantial training time reduction. However, similar to the sparse GP approach, forward variable selection requires multiple model trainings, partially offsetting theoretical computational gains. Nevertheless, training time remains significantly reduced compared to a full GP. These results establish the decomposed GP method with the BSS-ANOVA kernel as a viable option for accelerating training on this system.

One additional advantage that decomposed GPs have is that they can be updated incrementally with new, large batches of data. Using the previous Bayesian model, where the data used to train the model becomes stored in the posterior distribution of the weights of the basis functions, this posterior can become the new prior. This means that only new data needs to be presented to the model to update it, rather than needing to retrain on the entire data set, leading to faster training times. This can be a significant advantage in an online setting where data is arriving sequentially. The results of this incremental update can be seen in Table 6 and Figure 5.

**Figure 5:**
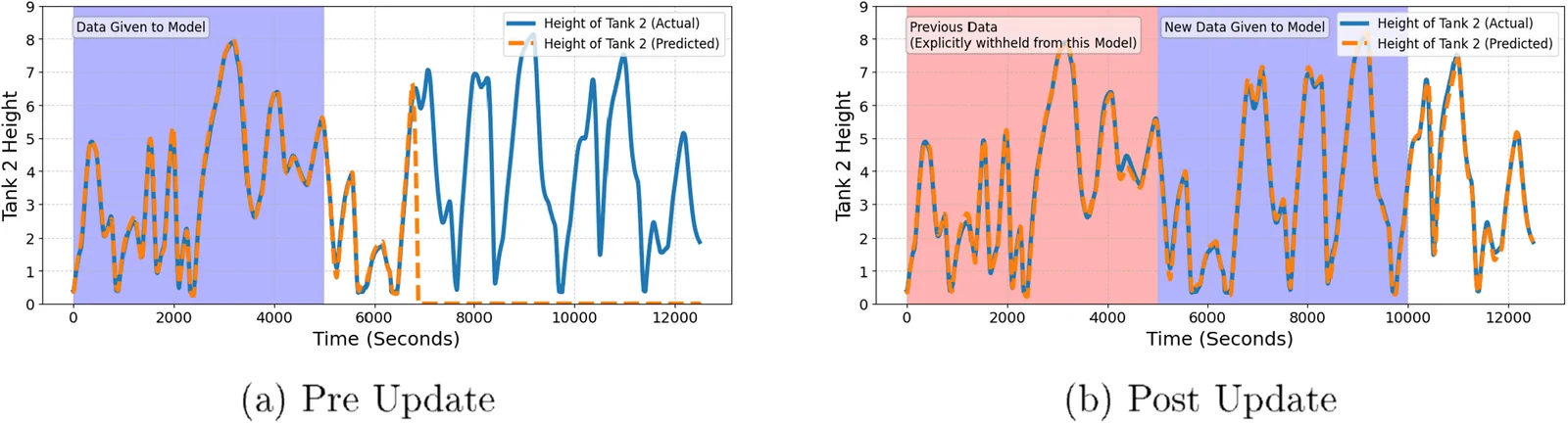
Incremental update comparison visuals.

**Table 6:** Incremental Update Comparison Metrics

Data and Prior Given	MSE	Training Time (s)
0–5000 s with uninformed prior	3.8251 (Failure)	2.9
5000–10000 s with strong prior	0.0151	5.7

During the initial training period (0–5,000 s), the model failed to capture the full system dynamics. This failure likely resulted from sparse data coverage in the limited training set, causing the model to extrapolate beyond its training domain as the simulation progressed. However, after updating the model with the subsequent 5,000 s of data, using the previous model’s weight distributions as the prior, the model successfully captured the full system dynamics, incorporating new information while retaining previously learned patterns. Compared to training on the complete 10,000 s data set, this incremental approach produced slightly higher error due to the approximation of **β** sampling as normal distributions. Nevertheless, incremental training achieved approximately 2 s (about a 25%) reduction in training time relative to retraining on the whole data set.

### 4.2 Closed-Loop MPC Validation

The MPC was tuned (*N*_*c*_ = 3; *N*_*p*_ = 15; *Q* = 1; *R* = 10^–5^) to balance both process and computational performance. The *N*_*c*_ and *N*_*p*_ values were set following available heuristics^54^ with trial-and-error adjustments based on performance testing where shorter times were selected due to known hardware limitations of the experimental setup. The control penalty weight *R* was tuned to balance tracking performance against control effort, ensuring no offset was present. PID controller gains were manually tuned (*K*_*p*_ = 6; *K*_*i*_ = 0.4; *K*_*d*_ = 6) to achieve closed-loop stabilization within the 600 s window. The Gaussian process was trained using two, three, and five set points worth of PID data to evaluate the effectiveness of MPC performance with additional data. The resulting training trajectories are shown in Figure 6 and Table 7.

**Figure 6:**
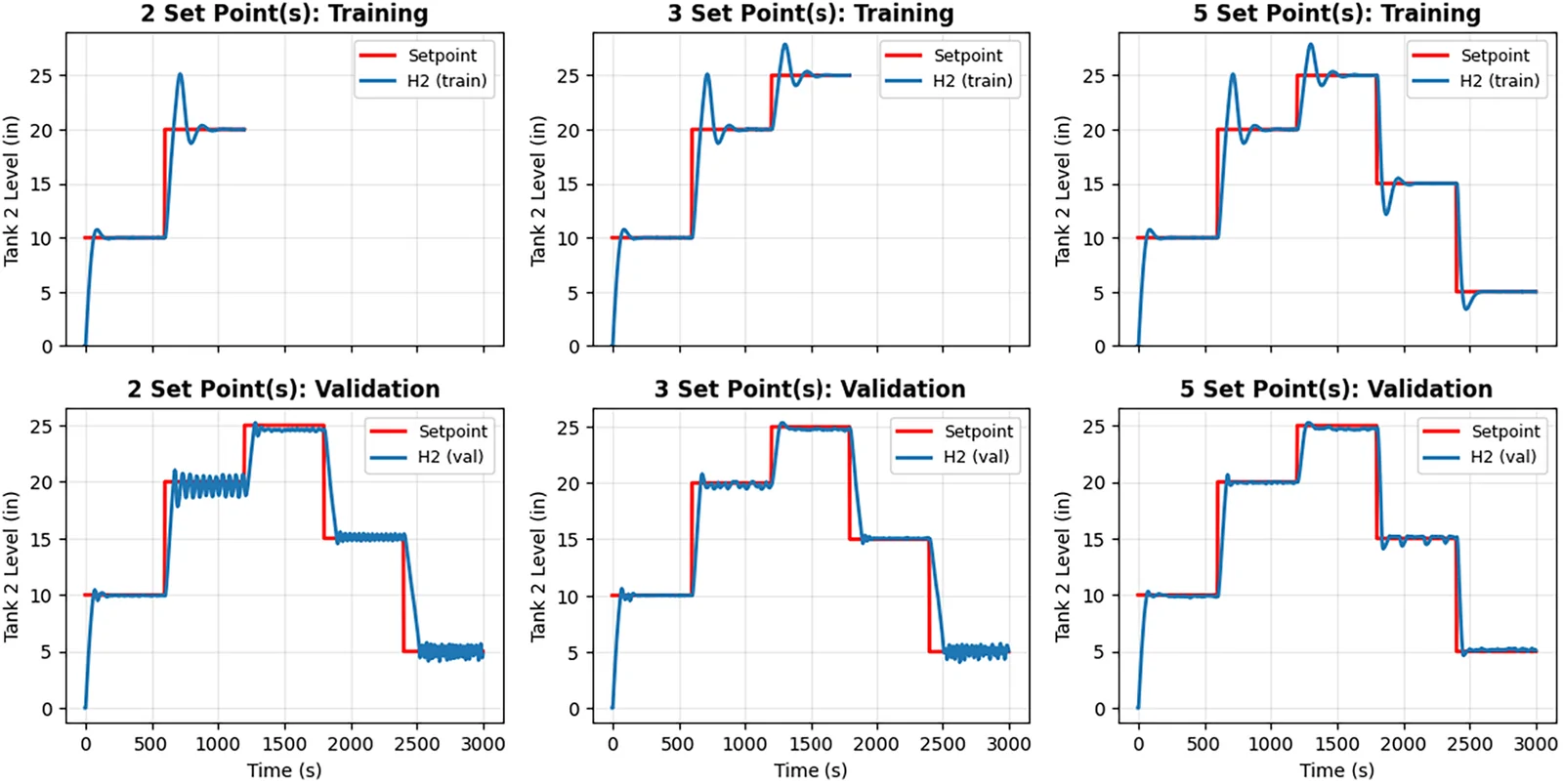
Training data and MPC performance for simulated cascaded tanks.

**Table 7:** MPC Validation Results

Number of PID Training Set points	MSE
2	0.2032
3	0.0642
5	0.0367

The results validate the effectiveness of the MPC with the considered states and the central hypothesis that augmenting the training data set improves MPC controller performance. The overall MSE for the measured output (height of the second tank) is computed by calculating the MSE for each set point following a 60 s transient period (corresponding to 10% of the total time following each set point change) to allow the system to reach steady state. This transient exclusion mitigates the influence of the large initial error following set point changes, which would otherwise dominate the overall MSE metric. The computed MSE values demonstrate that increasing the training data from two to three set points, and further to five set points, progressively improves overall MPC performance, thereby validating the benefit of model retraining.

### 4.3 Evaluation of Re-identification Metrics

For all evaluations presented in this section, a Gaussian process with an exponential kernel initially trained on data from the two set point PID controller case was used. Evaluation of the MPC controller used the 5 set point cycle as presented in the MPC validation. MPC simulations were executed on the Dolly Sods high-performance computing cluster (256 GB RAM, 2.6 GHz Processor) to ensure consistent model training times across all methods and allow for parallel assessment of metrics. The results are presented in Table 8 and Figure 7.

**Figure 7:**
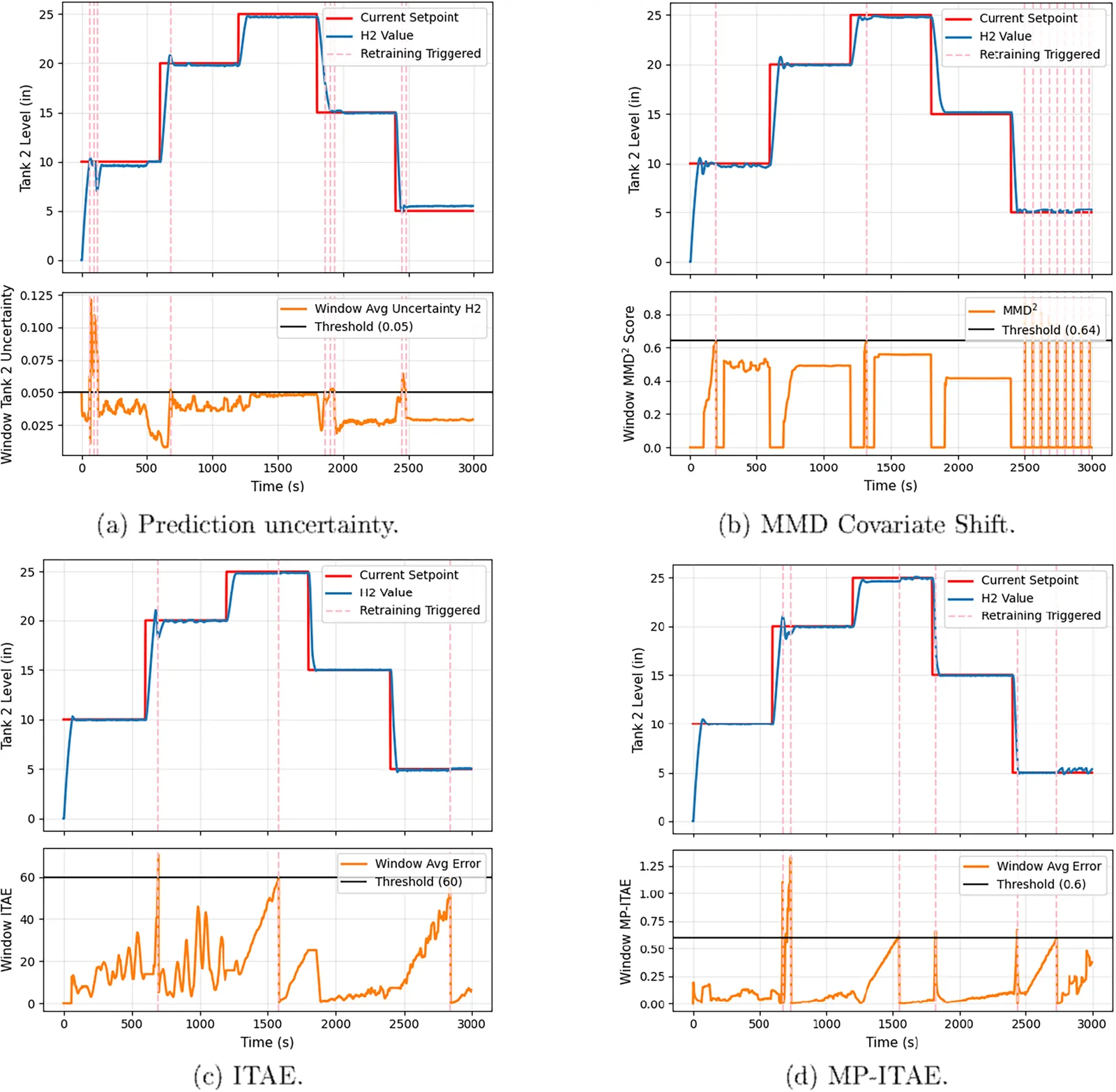
Comparison of four different re-identification metrics.

**Table 8:** Comparison of Re-identification Methods

Re-identification Method	MSE	Average Training Time (s)	Number of Training Events
Prediction Uncertainty	0.1496	4.75	9
MMD Covariate Shift	0.0362	8.49	11
ITAE	0.0099	10.23	3
MP-ITAE	0.0316	7.79	6

To implement the prediction uncertainty approach, several thresholds were tested, and an average uncertainty of 0.05 over a 30 s (10 sample) rolling window was selected as a representative threshold based on the metric’s baseline range and practical significance. If either prediction uncertainty for the first or second tanks dynamics passed the threshold, the model went through retraining. Upon retraining, all available data is used for training, including data from periods when uncertainty remained below the threshold.

The uncertainty based retraining approach yielded modest improvement over the baseline model shown in Figure 6. However, significant limitations emerged. Most notably, persistent offsets occurred at multiple set points. Because retraining is decoupled from control performance, the trigger remains inactive as long as prediction uncertainties stay below the threshold, regardless of tracking error. Additionally, several retraining events occur during set point transitions, indicating elevated uncertainty during these transients. However, steady-state tracking accuracy is the primary performance criterion. Since retraining events do not yield steady-state data at new set points, the integrated training data provides minimal benefit for steady-state region accuracy.

For the MMD covariate shift (evaluated based on MMD^2^), several measures were added to ensure robust operation of the metric. The target distribution was configured to be restricted to a 60 s window of recent data to assess exploration of new input regions. Several threshold values were tested, and an MMD^2^ threshold of 0.64 was selected as representative based on the distributional characteristics observed during initial exploratory simulations. Z-score normalization, computed from source data statistics, equalized the weighting of the three inputs. *K*-means clustering limited the source data to 100 representative samples, preventing redundant data from disproportionately influencing the metric calculation.

The MMD covariate shift based approach achieves substantial improvement over the baseline with no retraining. However, notable deficiencies appear. Persistent offsets occur at 25 and 15 in. set points. Interestingly, the 15 in. set point exhibits minimal MMD^2^ values despite having no direct training data, likely because of the multimodal nature of the data distribution that results in deceptively low MMD^2^ scores at interpolated regions. A core limitation of MMD-based re-identification is that the input data is inherently multimodal, with large clusters concentrated around particular set points. Although data filtering partially mitigated this issue, the multimodal structure remained a persistent source of spurious re-identification triggers, such as those seen at the 5 in. set point where repeated retraining cycles are triggered but fail to reduce the MMD^2^ below the threshold. While each iteration marginally improves the MMD^2^ metric, performance degradation occurs, demonstrating that distributional similarity does not guarantee control performance.

For the ITAE investigation, several threshold values were tested, and model retraining was configured to trigger when the average ITAE over a 30 s rolling window exceeds a threshold of 60, corresponding to an acceptable steady-state tolerance of approximately 0.1 in. at the maximum time scale. The ITAE time is reset to zero following each set point change and retraining event. A 60 s delay is imposed after set point changes only to allow the system to reach steady state before error accumulation begins, as significant ITAE values appear during these times and unwanted retraining events occur before the system can reach set point. The ITAE based approach exhibits superior performance relative to other methods. This improvement is attributable to the direct coupling between the retraining trigger and actual control performance metric, MSE of the height of the second tank.

For the MP-ITAE approach, several threshold values were tested, and a threshold of 0.6 was selected as representative based on model prediction accuracy requirements during initial exploration. This approach successfully mitigated offsets at the 20 and 25 in. set points through timely retraining. However, a notable deficiency occurred at the 5 in. set point. Despite achieving excellent control (within 0.05 in. of set point), retraining is triggered at approximately 2700 s due to model-measurement discrepancies. For this case, the MPC optimization problem predicts a slower system response than the simulated system. This means that the system is able to reach set point much quicker than anticipated. While this results in excellent control, the metric calculates a large error in the discrepancy between the model and actual simulation data. This retraining degrades control performance, revealing a fundamental limitation that while models that perfectly reflect the system will always work, there exist imperfect models that are still able to successfully control the system.

All four retraining approaches improve baseline performance. However, each exhibits distinct limitations. Input-space methods (prediction uncertainty and MMD) fail to prevent steady-state offsets because they remain agnostic to control performance. Conversely, performance-based methods (ITAE and MP-ITAE) directly address control quality. ITAE requires a delay after set point changes to suppress spurious retraining during the approach to the set point, while MP-ITAE can trigger retraining when model accuracy degrades regardless of control performance quality. Given the premise that the MPC controller will exhibit directional correctness during set point transitions, the ITAE transient delay incurs minimal performance penalty. The fundamental distinction between these two criteria is that MP-ITAE directly quantifies plant-model mismatch and model validity, whereas ITAE reflects closed-loop tracking performance. While an accurate model representation would theoretically guarantee well-controlled behavior, the closed-loop simulation results suggest that enforcing strict model validity as a re-identification trigger can impose unnecessary computational overhead by initiating retraining when controller performance remains acceptable. Accordingly, ITAE was selected as the retraining trigger for subsequent studies because its objective, minimizing control tracking error, aligns directly with the primary performance goal.

### 4.4 Evaluation of GP Algorithms

Six total methods were evaluated, two from each of the three GP approximation categories. Simulations were performed on the Dolly Sods HPC Cluster (256 GB RAM, 2.6 GHz Processor). All methods initialize their baseline models using training data from two set points collected via PID control and had inputs normalized from 0 to 1. Table 9 summarizes the comparative performance metrics across all evaluated methods, including prediction accuracy (MSE), average training time per retraining event, and total number of training events during the validation experiment.

**Table 9:** Comparative Performance Metrics of GP Algorithms

Model	MSE	Average Training Time (s)	Number of Training Events
GP (Exponential Kernel)	0.0099	10.23	3
SGPs (*K*-Means Only)	0.0085	0.33	2
SGPs (MML)	0.1547	3.86	12
Localized GPs (3 Centroids)	0.0145	4.71	4
Localized GPs (6 Centroids)	0.0217	2.14	4
Decomposed GPs (with Uninformative Prior)	0.0046	1.62	1
Decomposed GPs (with Strong Prior)	0.0057	1.41	2

The SGP approach using only *k*-means clustering was tuned to use 200 inducing points that approximate the full training set, thereby constraining model training time to a fixed computational budget. The *k*-means only approach achieved substantial computational acceleration, reducing average training time from 10.23 to 0.33 s relative to full GP regression as shown in Figure 7c. Additionally, this model resulted in better control and less training events. This is possible because the fictitious points selected via this process may better generalize to the overall system. Results are presented in Figure 8a.

**Figure 8:**
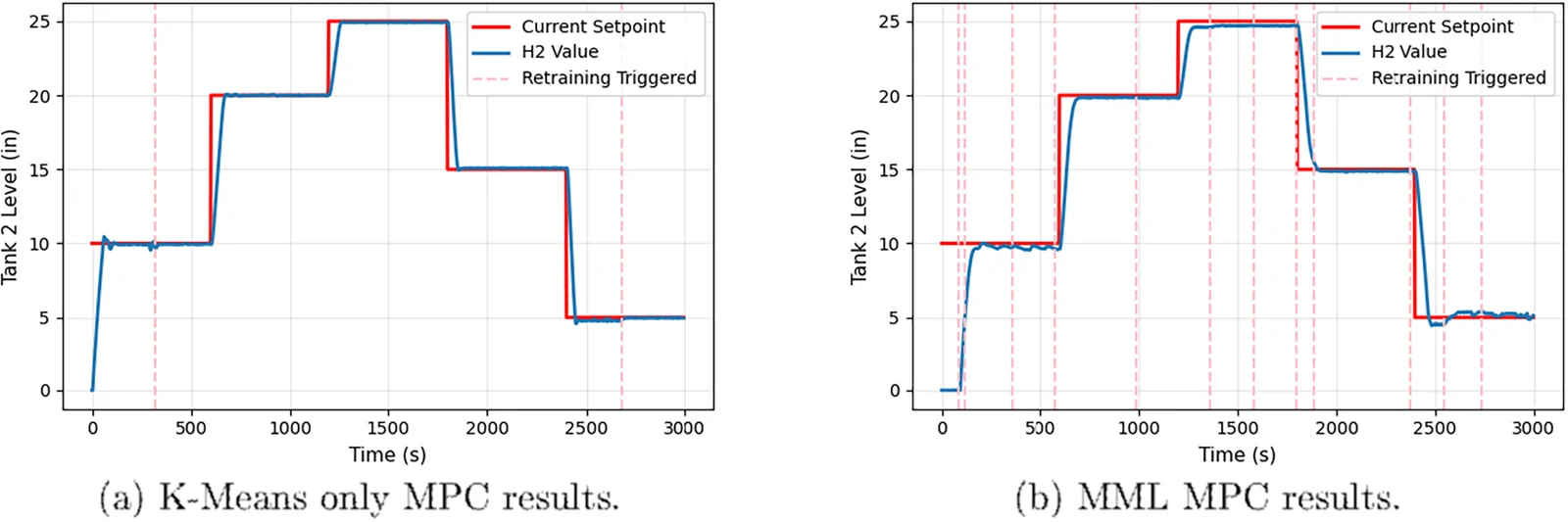
Sparse GP online simulation results.

The MML optimized approach exhibited significant performance degradation relative to *k*-means only selection. Notably, at initialization, the optimized inducing points predicted counterintuitive dynamics. With only a sparse number of data points in the empty-tank operating region, the inducing point optimization relocated points away from this region, resulting in extrapolatory predictions under those conditions for the initial model. This behavior arises because inducing points, when treated as optimization variables, migrate to regions that can violate the underlying system physics and data. After sufficient data accumulation in this region and subsequent retraining, the model recovered and was able to advance to the set point. This deficiency reveals a fundamental limitation of the optimization process of SGP methods that when training points are decoupled from actually observed data, the model risks overfitting to empirical observations while failing to generalize to true system dynamics. Results are shown in Figure 8b.

Two configurations of localized GPs were evaluated: 3-centroid and 6-centroid partitioning. Results are presented in Figures 9a,b. Both configurations achieved adequate closed-loop control performance. While the 6-centroid variant showed a higher MSE result (which could suggest overfitting), a conclusive determination on the training speed cannot be made due to the different timing of the model retrainings between the 3 and 6 centroid cases, meaning the trainings contained varying amounts of data points.

**Figure 9:**
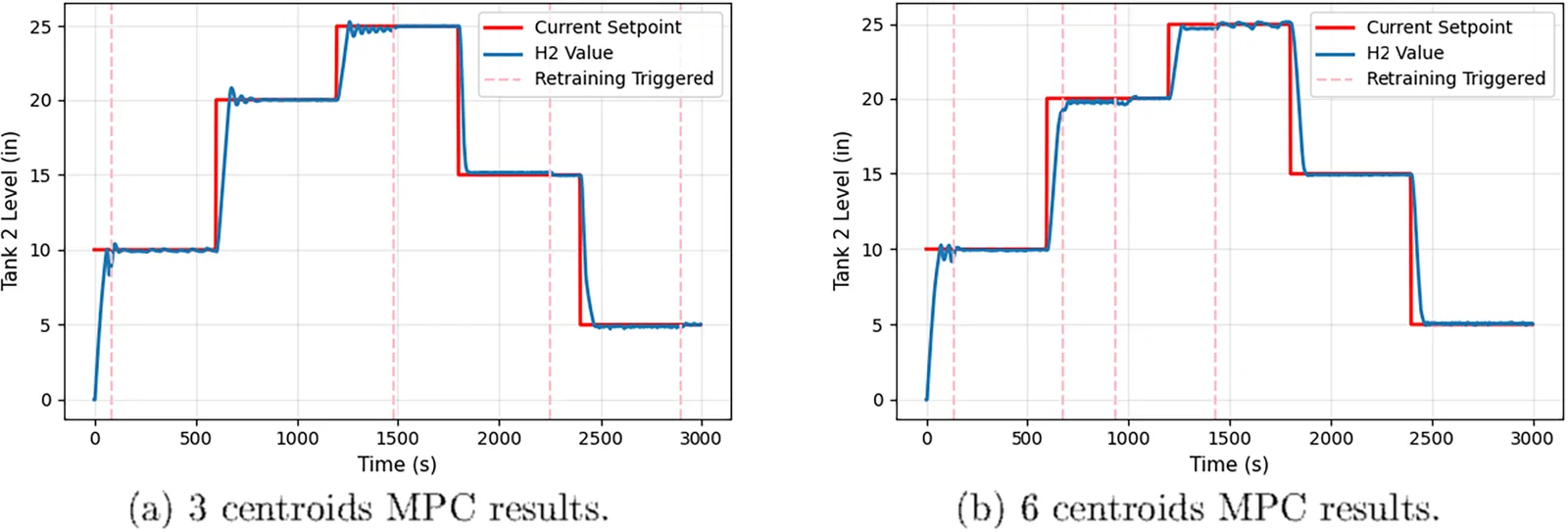
Localized GP online simulation results.

The results of the decomposed GP method using BSS-ANOVA basis functions and an uninformed prior can be seen in Figure 10a. The decomposed GP approach demonstrated remarkable generalization from limited training data. Only using the two-set point PID data, the model accurately captured system dynamics across the 10, 20, and part of the 25 in. set points. Performance metrics were superior relative to other methods with the lowest overall MSE and a 7-fold acceleration in training time compared to the standard GP regression. Minor tracking errors occurred only at the 25 in. set point (0.15 in. offset), which was corrected following retraining. Notably, the decomposed GP model exhibited the fastest transient response to set point changes among all methods, a characteristic partially obscured in other approaches due to ITAE calculation delays, indicating superior responsiveness to system dynamics.

**Figure 10:**
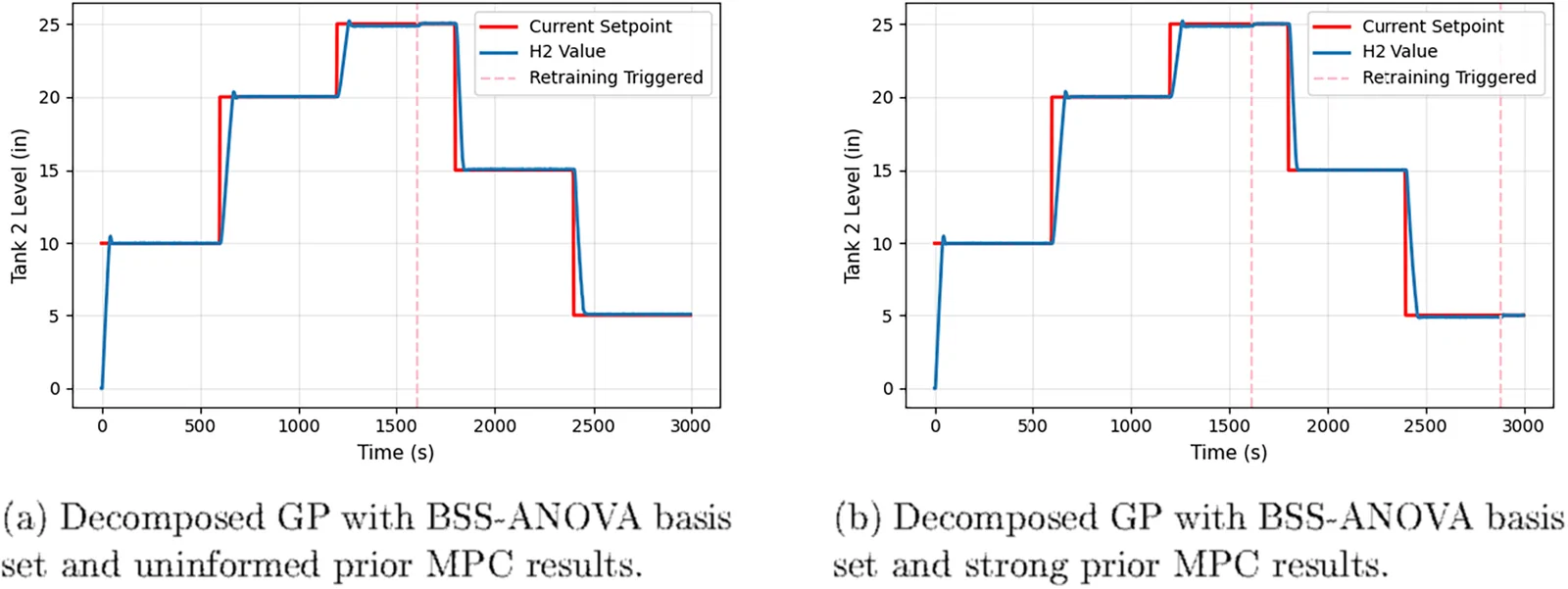
Decomposed GP online simulation results.

The results for incorporating strong priors on the model parameters during retraining are presented in Figure 10b. The strong prior-informed approach maintains excellent accuracy while enabling effective model refinement, as evidenced by successful retraining corrections at 25 and 5 in. set points. Compared to the uninformed prior variant that utilizes all available training data simultaneously, the strong prior-based retraining incurs slight accuracy degradation and triggers one additional retraining event. Although marginally faster than uninformed prior training, this trade-off reveals a fundamental limitation. The assumption that model parameters follow a normal distribution implicitly truncates the posterior distribution, thereby excluding information that would otherwise be captured by training on the complete data set.

This section demonstrated multiple effective approaches for reducing Gaussian process training time. However, computational efficiency gains typically incur accuracy trade-offs. The *k*-means only SGP approach performs exceptionally well, achieving the fastest training time among all evaluated methods while maintaining acceptable model fidelity. Notably, this data reduction strategy is not exclusive to SGP formulations and may be generalized as a preprocessing technique for alternative training methodologies for big data applications. The decomposed GP approach demonstrated superior overall performance, achieving the lowest prediction error while simultaneously enabling substantial computational acceleration. A comparative analysis of models with and without strong priors revealed negligible differences in both prediction accuracy and average training time. For this application, the uninformed prior decomposed GP model is preferred due to the need for less model retraining and only marginal computational benefits associated with strong prior incorporation in this particular use case. Therefore, the uninformed prior decomposed GP was used in the experimental evaluations.

### 4.5 Evaluation of Stability and Recursive Feasibility

Following the selection of the decomposed GP approximation technique and ITAE-based retraining strategy, a comprehensive stability analysis was performed to validate the theoretical guarantees. The multiset point evaluation systematically applied eigenvalue-based stability analysis and terminal set design across the cascaded tanks operating range (5–25 in. in 5 in. increments). For each set point, equilibrium points were computed from the steady-state flow balance, the system was linearized to obtain Jacobian matrices, and the DARE was solved.

Stability margins were calculated for three models: the first-principles plant model based on Torricelli’s law, the initial decomposed GP model trained on two-set point PID data, and the final GP model after a single online update during MPC operation. The closed-loop eigenvalue spectra quantify local asymptotic stability, while terminal set dimensions characterize the operational feasibility at each set point. Results of the analyses are presented in Figures 11 and 12.

**Figure 11:**
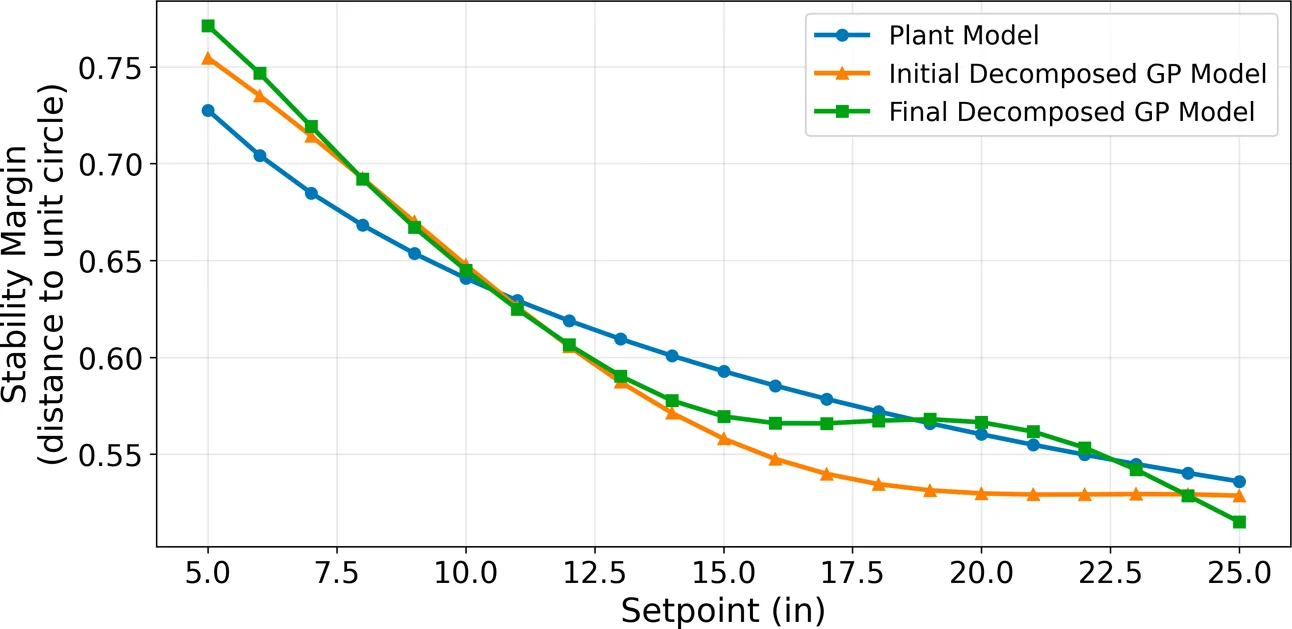
Discrete-time closed-loop eigenvalue trajectories across operating range for plant model and initial and final decomposed GP models.

**Figure 12:**
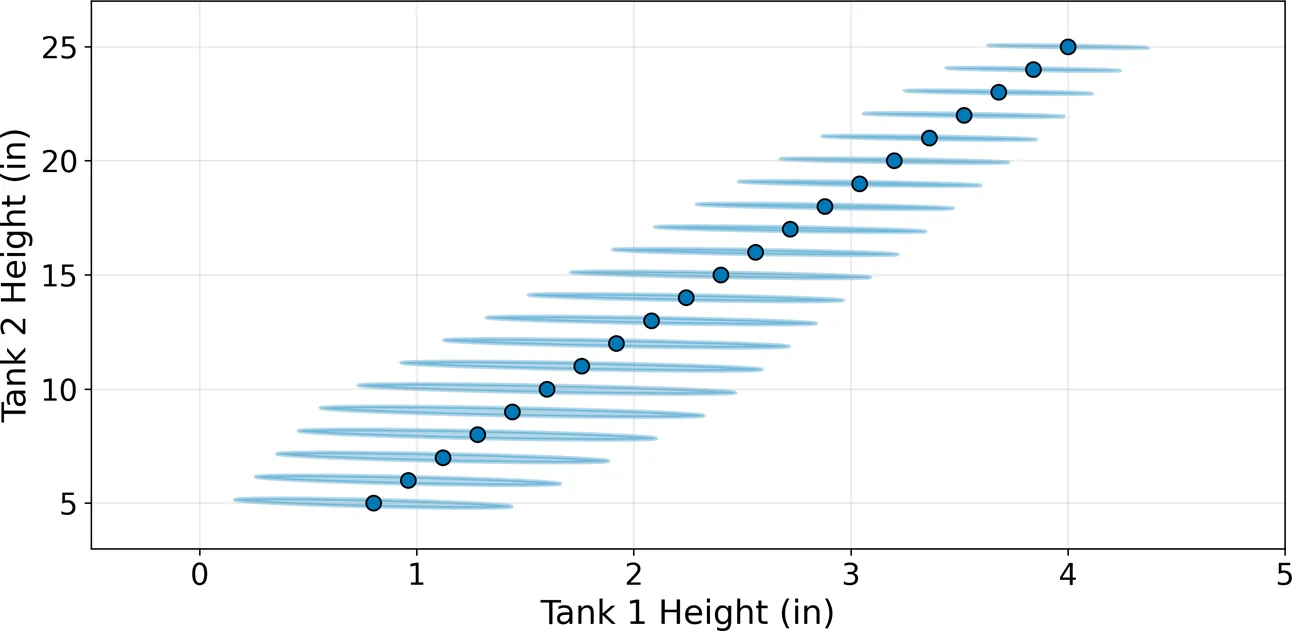
Terminal invariant sets across operating range for plant model.

The eigenvalue analysis confirms local asymptotic stability across the entire operating range for all three models. Stability margins, defined as the distance to the unit circle for discrete time implementation, range from 0.52 to 0.77, indicating well-damped closed-loop dynamics. The stability margins decrease with an increasing set point height, a consequence of the nonlinear Torricelli dynamics that produce weaker linearized damping at higher tank levels. This characteristic suggests that the controller exhibits more robust stability at lower operating points, with greater tolerance to gain variations and reduced sensitivity to model uncertainty.

The comparison between physical plant and GP model eigenvalues validates the fidelity of the data-driven surrogate models. After updating the GP model through online retraining, the stability margins converge toward those of the plant system at the 20 in. set point. However, small deviations do appear at the data sparse ends of the range.

Terminal set analysis reveals nonempty positively invariant regions at all evaluated set points, confirming that recursive feasibility can be guaranteed throughout the operating range. The smallest terminal set at 25 in. permits approximately ± 0.5 in. deviations in the height of tank 1, though the acceptable range for tank 2 heights decreases with increasing set point due to the increased outlet flow rate caused by Torricelli’s law. While these terminal sets ensure stability guarantees, should the invariant region areas want to be increased, the control penalty weight R could be enlarged to improve operational robustness. While terminal constraints are not explicitly enforced in the simulations presented in this analysis, the closed-loop trajectories of the decomposed GP-MPC reach steady-state operating points that lie within the computed terminal sets at each set point, suggesting that recursive feasibility could be formally guaranteed if the terminal constraints were imposed.

From a practical implementation perspective, these results provide confidence that the MPC optimization will remain feasible throughout operation provided the terminal constraints are enforced. The worst case closed-loop 95% settling time of approximately 12 s, validates the selection of the 45 s prediction horizon (*N*_*p*_ = 15 samples at 3 s intervals), ensuring sufficient predictive look-ahead for the MPC to be effective.

### 4.6 Experimental System Validation

The WVU Unit Operations Lab cascaded tanks setup was used as the real-world system to validate the previous results discussed. Computations were run on laptop level hardware (8 GB RAM, 1.6 GHz Processor) which was used for the MPC and connected to an Arduino to control the physical system. The same initial training method was used with PID data collected at 10 and 20 in. following the previously described methodology to train the MPC. The sampling frequency was set to 5 s to ensure that both model retraining and MPC optimization could be completed within each sampling interval. The retraining threshold was configured to an ITAE tolerance of $\frac{1}{3}$ in. at the maximum time scale, selected as a representative value that balances accuracy and retraining frequency. The results are shown in Table 10. Figure 13 presents the closed-loop control data comparing the GP approximation approach using the decomposed GP with the two hybrid modeling methodologies. Both hybrid methodologies outperformed the case with no physics integrated into the system.

**Figure 13:**
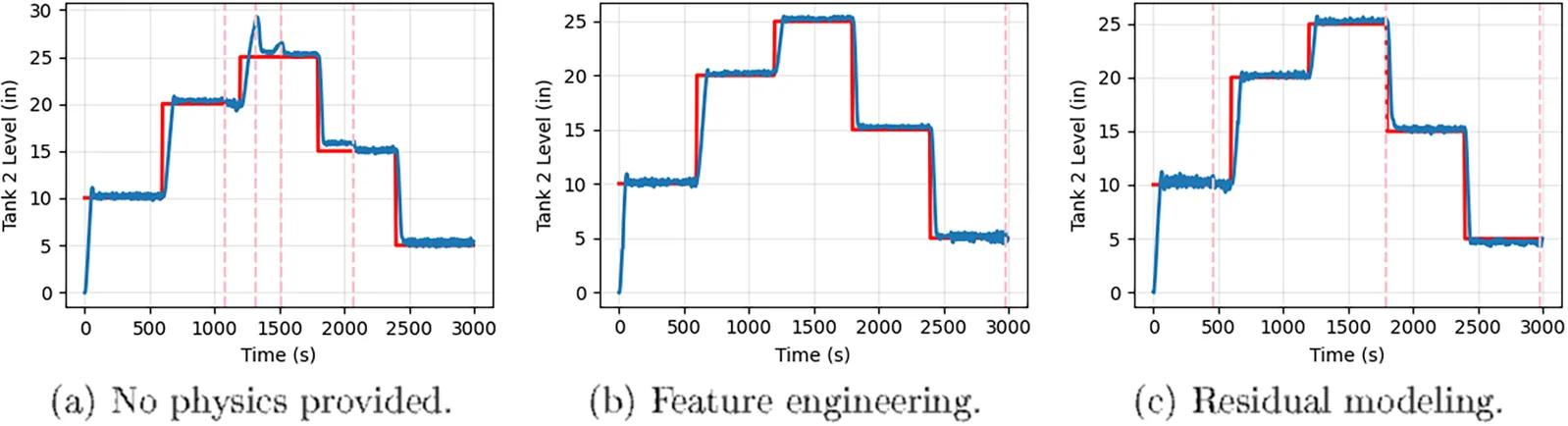
Results of experimental setup with hybrid machine learning.

**Table 10:** Comparative Performance of Hybrid Methodologies on Real-World System

Model	MSE	Average Training Time (s)	Number of Training Events
No physics provided	0.1883	2.85	4
Feature Engineering (Square Root Scaling)	0.0770	3.01	1
Residual Modeling	0.1155	3.90	3

The decomposed GP approach demonstrated adequate control performance when implemented with model retraining. The 10 in. set point is successfully controlled within tolerance solely using the initial training data. However, at the 20 in. set point (which had corresponding training data), the ITAE passed its training threshold at approximately 80% through the set point, requiring model retraining. The 25 in. set point exhibited significant overshoot, demonstrating poor generalization to unseen operating conditions, which is a marked departure from the simulation results and reflects the impact of real-world system nonidealities. This also shows one of the downsides of the delay implemented into the ITAE metric, which prevented the model from retraining during the transitionary period between set points, allowing for the large overshoot. Following two retraining events, however, control is achieved within tolerance. A similar situation occurs at the 15 in. set point, necessitating retraining due to significant initial offset.

The feature engineering approach yielded superior performance in comparison. Leveraging the same initial two set points of training data, the method successfully controlled the first four subsequent set points within the specified error tolerance, demonstrating effective generalization for this problem. The 5 in. set point is also controlled, though it slightly exceeds the tolerance threshold, triggering model retraining after 90% of the set point is complete.

The residual modeling approach yielded improved results compared to the base case. However, it exhibited distinct operational characteristics. Notably, the method demonstrates a tendency for on/off control behavior at the initial 10 in. set point, resulting in oscillations in the second tank’s height. However, this behavior is subsequently corrected following model retraining. Additionally, small but persistent offsets associated with the residual modeling approach caused the ITAE metric’s time-weighted component to accumulate sufficiently near the end of the 25- and 5-in. set points to trigger inefficient retrainings with minimal time left at those set point. Furthermore, the computational time of retraining is higher, attributed to the requirement of solving the additional optimization problem for the first-principles system during each retraining instance.

To analyze the residual modeling control action more in depth, Figure 14 illustrates the performance of the physics-based model in isolation on the initial PID training data.

**Figure 14:**
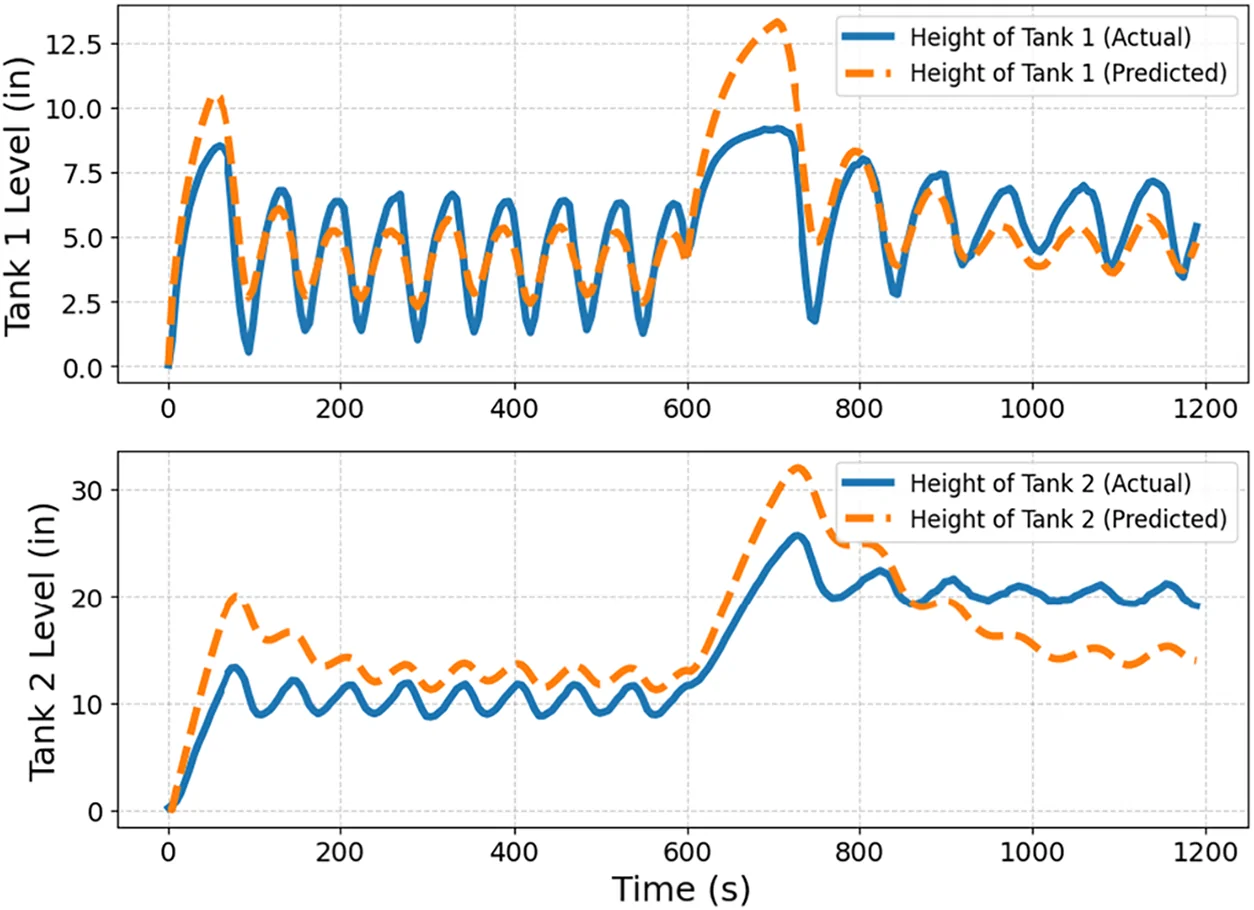
Performance of isolated physics-based model from residual model method.

While the Torricelli-based physics model captures the general oscillatory trends, the predicted magnitudes deviate from observations, and the long-term directional trends are inconsistent, suggesting the presence of unmodeled phenomena. To analyze the performance of the residual model, Figure 15a,15b compare the learned dynamics of the original dynamics captured by the traditional approaches versus the residual dynamics (observations minus physics predictions) targeted by the residual model approach.

**Figure 15:**
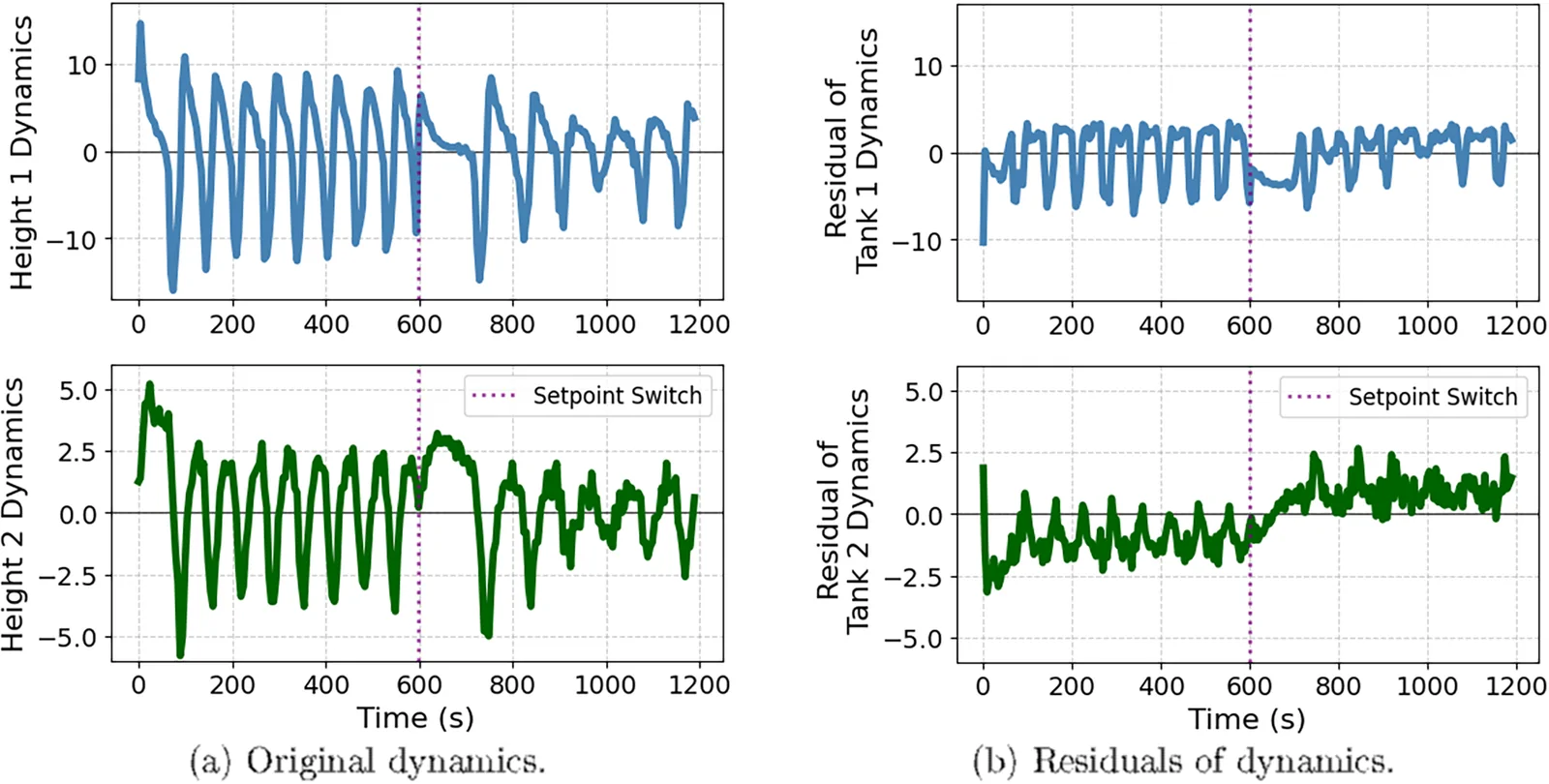
Comparison of dynamics and residuals of dynamics.

Examination of these figures reveals a significant reduction in the predictive range required of the decomposed GP model. Additionally, a comparison of the results indicates a fundamental shift in the underlying pattern for the Tank 2 height variable. The previously observed oscillatory behavior is substantially attenuated and replaced by a distinct offset. Residuals exhibit negative values prior to the set point change and positive values thereafter. This shift in the target variable for the GP approximations may increase susceptibility to overfitting noise due to the reduced signal-to-noise ratio. Consequently, modeling the details of the system dynamics can become more challenging.

### 4.7 Framework Limitations and Generalizability Considerations

While the proposed framework successfully identified high-performing model architectures and re-identification strategies for the cascaded tanks system, several important limitations must be acknowledged to properly bound the conclusions and guide future applications.

#### 4.7.1 System Complexity Bounds

The cascaded tanks system, while exhibiting nonlinear dynamics, represents a relatively low-dimensional control problem with continuous, smooth dynamics. The framework’s performance on systems with significantly higher dimensionality, strong state coupling, or discontinuous behavior remains an open research question. Systems with several state variables, hybrid discrete-continuous dynamics, or strong discontinuities may require additional model structure considerations beyond those evaluated in this work.

#### 4.7.2 Long-Term Data Management and Scalability

While the GP approximation methods address the computational cost of individual retraining events, the monotonic growth of training data over extended operational periods introduces practical challenges not fully addressed by this framework. Considering a typical process control application with 5-s sampling intervals, continuous operation generates approximately 6.3 million data points annually. Although the decomposed GP methodology demonstrates favorable $O\left({\mathrm{NP}}^{2}\right)$ scaling and incremental learning capabilities that partially mitigate this concern, industrial deployment over multiyear horizons will require explicit data management strategies.

Potential approaches include: (i) sliding window techniques that retain only recent data; (ii) selective data retention based on information content or novelty metrics; and (iii) periodic data set compression or summarization. The trade-offs between memory efficiency, historical knowledge retention, and retraining performance represent an important area for future investigation. The current framework does not prescribe specific data management policies, and optimal strategies will likely be application-dependent.

#### 4.7.3 Theoretical Guarantees versus Empirical Performance

The stability analysis framework presented in Section 2.7 provides theoretical tools for assessing local stability via linearization and ensuring recursive feasibility through terminal constraints. However, the model selection and re-identification trigger optimization process remains fundamentally empirical. The eigenvalue-based stability criterion provides a preliminary check for safe operation and can serve as a model acceptance check, but it does not guarantee optimal closed-loop performance or robustness to unmodeled disturbances. Additional analysis, such as the implementation of robust MPC architectures may be needed to more comprehensively assess systems of interest.

For the cascaded tanks system, ITAE-triggered retraining with decomposed GPs yielded superior performance. However, these specific architectural choices may not generalize to systems with different dynamics, constraint structures, operating regimes, or performance objectives. The framework provides a systematic methodology for identifying suitable choices through progressive validation (offline → simulation →hardware), but it does not eliminate the need for system-specific evaluation.

#### 4.7.4 Re-identification Threshold Selection and Validation Best Practices

The threshold values reported in this work were selected through preliminary testing on exploratory simulation trajectories to demonstrate the qualitative behavior and relative performance of different re-identification metrics. While this approach is suitable for methodological comparison and proof-of-concept demonstration, rigorous deployment in production environments should follow more structured validation practices to avoid potential bias.

Specifically, threshold selection for deployment should use separate validation trajectories that are distinct from the final evaluation trajectories used to report performance metrics. This follows standard machine learning practices of maintaining train/validation/test splits to prevent data leakage. For simulation-based validation, this entails generating multiple diverse operating scenarios (starting tank heights and cycling set point routines for the cascaded tanks example) that span the expected operational envelope, splitting these into independent validation and test sets, tuning thresholds on the validation set, and reporting final performance exclusively on the held-out test trajectories. Absolute performance values and optimal threshold magnitudes will be system-specific and should be determined through appropriate validation procedures when deploying the framework to new applications.

#### 4.7.5 Scope of Applicability

Based on the results obtained, the framework is expected to be most beneficial for processes characterized by (i) low dimensionality; (ii) operational time horizons and sampling rates yielding manageable data accumulation; and (iii) availability of initial characterization data for offline model validation. Processes with well-established first-principles models may benefit more from hybrid modeling approaches that incorporate physical structure, as demonstrated in Section 4.6, rather than purely data-driven methods.

These limitations do not diminish the framework’s utility but rather define its appropriate application domain and identify directions for future research, including extension to high-dimensional systems, development of principled data management strategies, integration with robust MPC formulations that explicitly leverage GP uncertainty quantification, and formalization of threshold selection procedures for production deployment.

## 5 Conclusions and Future Work

This research presented a framework for identifying accurate and computationally efficient GP approximations for the use in MPC systems through the use of historical data, online simulations, and hybrid modeling approaches. For the motivating example of a cascaded tanks experiment, it was demonstrated that a highly performant model architecture for the MPC can quickly be determined with minimal experimental effort. The results of this framework showed that a decomposed GP approximation technique with feature engineering provided superior performance for controlling a real-world system. ITAE and MP-ITAE were found to be the superior retraining metrics, showing that either system control performance or model accuracy need to be considered when determining when to retrain a model. Other complementary techniques were also found to be effective for training GPs with large data sets, such as *k*-means clustering for reducing information-poor data. Future work includes evaluating data management and reduction techniques and integrating robust or stochastic MPC formulations that explicitly leverage GP uncertainty quantification for constraint tightening and risk-aware optimization.

## Supplementary Material



## Data Availability

The supporting code and data for this article can be found online at the following GitHub site: https://github.com/michaelwfouts/Scaling-Gaussian-Processes-for-MPCs-with-Online-Updates.

## Notes

Derivation of training decomposed Gaussian process approximations with strong priors, focusing on the forward variable selection approach, considering cases when no new terms and new terms need to be added (PDF)
