# Supplementary material for: Scaling Gaussian Processes for Deterministic Model Predictive Control with Online Updates: A Framework for Model Selection and Re-identification in Process Control
Source: Ind Eng Chem Res. 2026 Aug 14;65(33):17897–917. doi: 10.1021/acs.iecr.6c01228 (PMC13523696; doi:10.1021/acs.iecr.6c01228)
Supplement: Supplementary file 1 [file ie6c01228_si_001.pdf]

Supporting Information

Scaling Gaussian Processes for Deterministic Model Predictive Control with Online Updates: A Framework for Model Selection and Re-identification in Process Control

Michael W. Fouts,† David S. Mebane,‡ and Fernando V. Lima*,†

†*Department of Chemical and Biomedical Engineering, West Virginia University, West Virginia, USA, 26506*

‡*Department of Mechanical, Materials and Aerospace Engineering, West Virginia University, West Virginia, USA, 26506*

E-mail: fernando.lima@mail.wvu.edu

The supporting information includes information on the derivation of training decomposed Gaussian process approximations with strong priors, focusing on the forward variable selection approach, considering cases when no new terms and new terms need to be added.

S1 Decomposed GP with Strong Prior Derivation

Decomposed GPs have already been shown to be effective for an uninformed model.[1] Part of this work is expanding on this idea to incorporate the prior of previously trained models as a strong prior to reduce the training time. This section derives the explicit conditional posteriors for all parameters, details their implementation within the blocked Gibbs framework for model updates, both with and without new terms.

S1.1 Derivation with No New Terms

First, the case where no new terms are added to the model will be evaluated. The posterior distribution of the parameters given the data can be expressed using Bayes' theorem as follows, with the relationship shown in Equation S1:

$$\Omega(\theta_0, \sigma^2, \tau^2 \mid y) \propto \mathcal{L}(y \mid \theta_0, \sigma^2, \tau^2) \cdot\, P(\theta_0 \mid \mu_O, \Sigma_O) \cdot\, P(\sigma^2 \mid a, b) \cdot\, P(\tau^2 \mid a_\tau, b_\tau) \tag{S1}$$

where y represents the observed data, θ_0 is the vector of model parameters with subscript 0 representing already existing model parameters, σ^2 is the observation error variance, and τ^2 is the prior variance for the parameters. The likelihood $\mathcal{L}(y \mid \theta_0, \sigma^2, \tau^2)$ is assumed to follow a normal distribution, the prior for θ_0 is assumed as a normal distribution, and σ^2 and τ^2 are specified as inverse gamma distributions.

The ultimate goal is to solve for the posterior distributions of θ_0, σ^2, and τ^2, of which, each is defined by their respective parameters (μ and Σ for normal distributions and a and b for inverse gamma distributions) using a Gibbs Sampling approach. This involves holding two of the parameters constant while solving for the third. Beginning with the full posterior distribution and expanding the probabilities on the right-hand side knowing the likelihood and theta prior are normal distributions and the error variance and theta prior variance are inverse gamma distributions, the following is obtained in Equation S2:

$$
\begin{aligned}
\Omega(\theta_0, \sigma^2, \tau^2 \mid y) \propto\ & (\sigma^2)^{-n/2} \exp\left[-\frac{1}{2\sigma^2}(y - X_0\theta_0)^\top (y - X_0\theta_0)\right] \\
& (\sigma^2\tau^2)^{-p_0/2}\, |\Sigma_0|^{-1/2}\ \exp\left[-\frac{1}{2\sigma^2\tau^2}(\theta_0 - \mu_0)^\top \Sigma_0^{-1}(\theta_0 - \mu_0)\right] \\
& (\sigma^2)^{-a-1}\ \exp\left(-\frac{b}{\sigma^2}\right) \\
& (\tau^2)^{-a_\tau-1}\ \exp\left(-\frac{b_\tau}{\tau^2}\right)
\end{aligned}
\tag{S2}
$$

where n is the number of observations and p_0 is the number of parameters (or basis functions) in θ_0, Σ_0 is the prior covariance matrix for θ_0, μ_0 is the prior mean for θ_0, and a, b, a_τ, and b_τ are the hyperparameters for the inverse gamma distributions.

Gibbs Sampling Part 1: Solving for θ_0 holding σ^2 and τ^2 constant

To calculate the mean and covariance matrix of the posterior distribution, using Gibbs sampling, any terms that do not have θ_0 in them can be removed as they are incorporated into the proportionality since they are constant in this case. This gives the following in Equation S3.

$$
\begin{aligned}
\Omega(\theta_0, \sigma^2, \tau^2 \mid y) \propto\ & (\sigma^2)^{-n/2} \exp\left[-\frac{1}{2\sigma^2}(y - X_0\theta_0)^\top (y - X_0\theta_0)\right] \\
& (\sigma^2\tau^2)^{-p_0/2}\, |\Sigma_0|^{-1/2}\ \exp\left[-\frac{1}{2\sigma^2\tau^2}(\theta_0 - \mu_0)^\top \Sigma_0^{-1}(\theta_0 - \mu_0)\right]
\end{aligned}
\tag{S3}
$$

All terms in front of the exponential can additionally be absorbed by the proportionality. This simplifies to Equation S4:

$$
\Omega \propto (y - X_0\theta_0)^\top (y - X_0\theta_0) + \frac{1}{\tau^2}(\theta_0 - \mu_0)^\top \Sigma_0^{-1}(\theta_0 - \mu_0) \tag{S4}
$$

Multiplying out the terms and removing the terms that do not contain θ_0 yields Equation S5.

$$
\begin{aligned}
\Omega \propto\ & -y^\top X_0\theta_0 - \theta_0^\top X_0^\top y + \theta_0^\top X_0^\top X_0\theta_0 \\
& + \frac{1}{\tau^2}\left(\theta_0^\top \Sigma_0^{-1}\theta_0 - \theta_0^\top \Sigma_0^{-1}\mu_0\right) + \frac{1}{\tau^2}\left(-\mu_0^\top \Sigma_0^{-1}\theta_0\right)
\end{aligned}
\tag{S5}
$$

Combining like terms together yields Equation S6.

$$
\Omega \propto -y^\top X_0\theta_0 - \theta_0^\top X_0^\top y + \theta_0^\top (X_0^\top X_0 + \frac{1}{\tau^2}\Sigma_0^{-1})\theta_0 - \frac{1}{\tau^2}\left(\theta_0^\top \Sigma_0^{-1}\mu_0\right) + \frac{1}{\tau^2}\left(-\mu_0^\top \Sigma_0^{-1}\theta_0\right) \tag{S6}
$$

Given that the posterior distribution is expected to be a multivariate normal distribution, the covariance matrix and mean of the posterior distribution can be identified. Starting with the formula for a multivariate normal distribution in Equation S7.

$$
f(x|\mu^*, \sigma^2) = (\sigma^2)^{-k/2}\, |\Sigma_0^*|^{-1/2}\, \exp\left[-\frac{1}{2\sigma^2}(\theta_0 - \mu_0^*)^\top (\Sigma_0^*)^{-1}(\theta_0 - \mu_0^*)\right] \tag{S7}
$$

Note that superscript * notes a value for the posterior distribution. Again, knowing that only the terms that contain θ_0 need to be considered, the above equation can be rewritten and expanded to Equation S8.

$$
(\theta_0 - \mu_0^*)^\top (\Sigma_0^*)^{-1}(\theta_0 - \mu_0^*) = \theta_0^\top (\Sigma_0^*)^{-1}\theta_0 - \theta_0^\top (\Sigma_0^*)^{-1}\mu_0^* - (\mu_0^*)^\top (\Sigma_0^*)^{-1}\theta_0 \tag{S8}
$$

Aligning this with Equation S6, the following relationships for the posterior covariance matrix can be identified in Equation S9.

$$
(\Sigma_0^*)^{-1} = \left(X_0^\top X_0 + \frac{1}{\tau^2}\Sigma_0^{-1}\right) \tag{S9}
$$

Considering the other terms of Equation S6 and grouping by the position of the θ_0 term, the contribution to the linear term is shown in Equation S10.

$$
\begin{aligned}
&\theta_0^\top \left(-X_0^\top y - \frac{1}{\tau^2}\Sigma_0^{-1}\mu_0\right) \\
&\quad + \left(-y^\top X_0 - \frac{1}{\tau^2}\mu_0^\top \Sigma_0^{-1}\right)\theta_0
\end{aligned} \tag{S10}
$$

Either grouping may be used to solve for μ_0^*. Selecting the grouping with θ_0 at the beginning gives Equation S11.

$$
-\theta_0^\top \left(\Sigma_0^*\right)^{-1} \mu_0^* = \theta_0^\top \left(-X_0^\top y - \frac{1}{\tau^2}\Sigma_0^{-1}\mu_0\right) \tag{S11}
$$

Solving for μ_0^* in Equation S12 gives the posterior mean.

$$
\mu_0^* = \Sigma_0^* \left(X_0^\top y + \frac{1}{\tau^2}\Sigma_0^{-1}\mu_0\right) \tag{S12}
$$

This provides an explicit solution for the mean and covariance parameters of the posterior distribution of the θ values.

Gibbs Sampling Part 2: Solving for σ^2 keeping θ_0 and τ^2 constant

Given that the resulting posterior is expected to follow an inverse-gamma distribution of the form in Equation S13.

$$
f(x; a, b) = \frac{b^a}{\Gamma(a)} x^{-a-1} \exp(-b/x) \tag{S13}
$$

Knowing that x is σ^2 for this derivation, the objective is to determine the parameters a^* and b^* for the posterior. Starting with Bayes' theorem, the terms that depend on σ^2 are shown explicitly in Equation S14.

$$
\begin{aligned}
\Omega \propto & (\sigma^2)^{-n/2} \exp\left[-\frac{1}{2\sigma^2}(y - X_0\theta_0)^\top (y - X_0\theta_0)\right] \\
& (\sigma^2\tau^2)^{-p_0/2} \left|\Sigma_0\right|^{-1/2} \exp\left[-\frac{1}{2\sigma^2\tau^2}(\theta_0 - \mu_0)^\top \Sigma_0^{-1}(\theta_0 - \mu_0)\right] \\
& (\sigma^2)^{-a-1} \exp\left(-\frac{b}{\sigma^2}\right)
\end{aligned} \tag{S14}
$$

Combining the exponents of the pre-exponential form and comparing to the inverse-gamma distribution form yields Equation S15.

$$
\begin{aligned}
-a^* - 1 &= -\frac{n}{2} - \frac{p_0}{2} - a - 1 \\
a^* &= \frac{n + p_0}{2} + a
\end{aligned} \tag{S15}
$$

To solve for b^*, noting that for this term, the only relevant terms are those inside the exponential, Equation S16 follows.

$$
b^* = \frac{1}{2}(y - X_0\theta_0)^\top (y - X_0\theta_0) + \frac{1}{2\tau^2}(\theta_0 - \mu_0)^\top \Sigma_0^{-1}(\theta_0 - \mu_0) + b \tag{S16}
$$

Expanding b^* gives Equation S17.

$$
\begin{aligned}
b^* = & \frac{1}{2}(y^\top y - y^\top X_0\theta_0) \\
& + \frac{1}{2}(-\theta_0^\top X_0^\top y + \theta_0^\top X_0^\top X_0\theta_0) \\
& + \frac{1}{2\tau^2}(\theta_0^\top \Sigma_0^{-1}\theta_0 - \theta_0^\top \Sigma_0^{-1}\mu_0) \\
& + \frac{1}{2\tau^2}(-\mu_0^\top \Sigma_0^{-1}\theta_0 + \mu_0^\top \Sigma_0^{-1}\mu_0) \\
& + b
\end{aligned} \tag{S17}
$$

This provides the explicit forms for calculating both the a^* and b^* parameters for the σ^2 inverse gamma distribution.

Gibbs Sampling Part 3: Solving for τ^2 keeping θ_0 and σ^2 constant

Similarly to σ^2, the posterior distribution for τ^2 can be derived assuming it takes the form of an inverse-gamma distribution. Solving for a_τ^* and b_τ^*, the final answers are as follows in Equations S18 and S19.

$$a_\tau^* = \frac{p_0}{2} + a_\tau \tag{S18}$$

$$\begin{aligned} b_\tau^* =& \frac{1}{2\sigma^2}(\theta_0^\top \Sigma_0^{-1}\theta_0 - \theta_0^\top \Sigma_0^{-1}\mu_0) \\ &+ \frac{1}{2\sigma^2}(-\mu_0^\top \Sigma_0^{-1}\theta_0 + \mu_0^\top \Sigma_0^{-1}\mu_0) \\ &+ b_\tau \end{aligned} \tag{S19}$$

S1.2 Derivation of Update with New Terms

Similar to the case with no new terms, the posterior distribution of the parameters given the data when new terms are added to the FoKL model via forward variable selection can be derived by adding in a term to consider the prior of the new terms. The posterior distribution can be expressed using Bayes' theorem as follows in Equation S20.

$$\Omega(\theta_0, \sigma^2, \tau^2 \mid y) \propto \mathcal{L}(y \mid \theta_N, \theta_0, \sigma^2, \tau^2) \cdot P(\theta_N \mid \mu_N, \Sigma_N) \cdot P(\theta_0 \mid \mu_O, \Sigma_O) \cdot P(\sigma^2 \mid a, b) \cdot P(\tau^2 \mid a_\tau, b_\tau) \tag{S20}$$

Expanding into the individual distributions yields Equation S21.

$$
\begin{aligned}
\Omega \propto & \left(\sigma^2\right)^{-n/2} \exp\left[-\frac{1}{2\sigma^2}(y - X_0\theta_0 - X_N\theta_N)^\top(y - X_0\theta_0 - X_N\theta_N)\right] \\
& \left(\sigma^2\tau^2\right)^{-p_N/2} \exp\left[-\frac{1}{2\sigma^2\tau^2}\,\theta_N^\top\theta_N\right] \\
& \left(\sigma^2\right)^{-p_0/2}\ |\Sigma_0|^{-1/2}\ \exp\left[-\frac{1}{2\sigma^2}(\theta_0 - \mu_0)^\top\Sigma_0^{-1}(\theta_0 - \mu_0)\right] \\
& \left(\sigma^2\right)^{-a-1} \exp\left(-\frac{b}{\sigma^2}\right) \\
& \left(\tau^2\right)^{-a_\tau-1} \exp\left(-\frac{b_\tau}{\tau^2}\right)
\end{aligned}
\tag{S21}
$$

To solve for the distribution parameters, this follows a similar process to that of the previous section where, via Gibbs Sampling, each set of distribution parameters is individually updated while holding the others constant.

Gibbs Sampling Part 1: Solving for θ_N holding θ_0, σ^2, and τ^2 constant

To calculate the mean of the θ_N terms, only the terms with θ_N need to be taken into consideration since the rest can be rolled into the proportionality.

$$
\begin{aligned}
\Omega \propto & (\sigma^2)^{-n/2} \exp\left[-\frac{1}{2\sigma^2}(y - X_0\theta_0 - X_N\theta_N)^\top(y - X_0\theta_0 - X_N\theta_N)\right] \\
& (\sigma^2\tau^2)^{-p_N/2} \exp\left[-\frac{1}{2\sigma^2\tau^2}\theta_N^\top\theta_N\right]
\end{aligned}
\tag{S22}
$$

The terms in front can now be taken out since they do not affect θ or μ, and the natural log of the terms can be taken and $-\frac{1}{2\sigma^2}$ removed.

$$
\Omega \propto (y - X_0\theta_0 - X_N\theta_N)^\top(y - X_0\theta_0 - X_N\theta_N) + \frac{1}{\tau^2}\theta_N^\top\theta_N \tag{S23}
$$

Multiplying out the terms gives the expanded quadratic form in Equation S24:

$$
\begin{aligned}
\Omega \propto &+ y^\top y - y^\top X_0\theta_0 - y^\top X_N\theta_N \\
&- \theta_0^\top X_0^\top y + \theta_0^\top X_0^\top X_0\theta_0 + \theta_0^\top X_0^\top X_N\theta_N \\
&- \theta_N^\top X_N^\top y + \theta_N^\top X_N^\top X_0\theta_0 + \theta_N^\top X_N^\top X_N\theta_N \\
&+ \frac{1}{\tau^2}\theta_N^\top\theta_N
\end{aligned}
\tag{S24}
$$

Combining terms to match the form of a multivariate normal distribution yields Equation S25.

$$
\begin{aligned}
\Omega \propto &+ y^\top y - y^\top X_0\theta_0 - y^\top X_N\theta_N \\
&- \theta_0^\top X_0^\top y + \theta_0^\top X_0^\top X_0\theta_0 + \theta_0^\top X_0^\top X_N\theta_N \\
&- \theta_N^\top X_N^\top y + \theta_N^\top X_N^\top X_0\theta_0 \\
&+ \theta_N^\top \left(X_N^\top X_N + \frac{1}{\tau^2}I\right)\theta_N \quad \rightarrow \quad \theta_N^\top(\Sigma_N^*)^{-1}\theta_N
\end{aligned}
\tag{S25}
$$

This suggests an explicit form of the posterior covariance matrix. Solving for the mean vector by factoring out the $\theta_N^\top$ term gives Equation S26.

$$
\theta_N^\top\left(-X_N^\top y + X_N^\top X_0\theta_0\right) \tag{S26}
$$

This aligns with the form of a standard multivariate normal distribution, as shown in Equation S27.

$$
-\theta_N^\top\left(\Sigma_N^*\right)^{-1}\mu_N^* = \theta_N^\top\left(-X_N^\top y + X_N^\top X_0\theta_0\right) \tag{S27}
$$

Therefore, the posterior for θ_N is multivariate normal with:

$$
\Sigma_N^* = \left(X_N^\top X_N + \frac{1}{\tau^2}I\right)^{-1} \tag{S28}
$$

$$\mu_N^* = \Sigma_N^* \left(X_N^\top y - X_N^\top X_0 \theta_0 \right) \tag{S29}$$

Gibbs Sampling Part 2: Solving for θ_0 holding θ_N, σ^2, and τ^2 constant

Solving for θ_0 follows a similar process. The posterior for θ_0 is multivariate normal, with covariance and mean given in Equations S30 and S31.

$$\Sigma_0^* = \left(X_0^\top X_0 + \Sigma_0^{-1} \right)^{-1} \tag{S30}$$

$$\mu_0^* = \Sigma_0^* \left(X_0^\top y - X_0^\top X_N \theta_N + \Sigma_0^{-1} \mu_0 \right) \tag{S31}$$

Gibbs Sampling Part 3: Solving for σ^2 holding θ_N, θ_0, and τ^2 constant

Assuming σ^2 takes the form of an inverse gamma distribution, the shape and scale parameters of the posterior distribution are as follows in Equations S32 and S33.

$$a^* = \frac{n}{2} + \frac{p_N + p_0}{2} + a \tag{S32}$$

$$b^* = \frac{1}{2}(y - X_0\theta_0 - X_N\theta_N)^\top (y - X_0\theta_0 - X_N\theta_N) + \frac{1}{2\tau^2}\theta_N^\top \theta_N + \frac{1}{2}(\theta_0 - \mu_0)^\top \Sigma_0^{-1} (\theta_0 - \mu_0) + b \tag{S33}$$

Gibbs Sampling Part 4: Solving for τ^2 holding θ_N, θ_0, and σ^2 constant

Similar to σ^2, solving for the posterior distribution of τ^2 yields the following in Equations S34 and S35.

$$a_\tau^* = \frac{p_N}{2} + a_\tau \tag{S34}$$

$$b_\tau^* = \frac{1}{2\sigma^2}\theta_N^\top \theta_N + b_\tau \tag{S35}$$

References

(1) Hayes, K.; Fouts, M. W.; Baheri, A.; Mebane, D. S. Forward Variable Selection Enables Fast and Accurate Dynamic System Identification with Karhunen-Loève Decomposed Gaussian Processes. *PLOS ONE* **2024**, *19*, e0309661.
